# Nucleolin Regulates Phosphorylation and Nuclear Export of Fibroblast Growth Factor 1 (FGF1)

**DOI:** 10.1371/journal.pone.0090687

**Published:** 2014-03-04

**Authors:** Torunn Sletten, Michal Kostas, Joanna Bober, Vigdis Sorensen, Mandana Yadollahi, Sjur Olsnes, Justyna Tomala, Jacek Otlewski, Malgorzata Zakrzewska, Antoni Wiedlocha

**Affiliations:** 1 Department of Biochemistry, Institute for Cancer Research, The Norwegian Radium Hospital, Oslo University Hospital, Oslo, Norway; 2 Centre for Cancer Biomedicine, Faculty of Medicine, University of Oslo, Norway; 3 Department of Protein Engineering, Faculty of Biotechnology, University of Wroclaw, Wroclaw, Poland; 4 Department of Protein Biotechnology, Faculty of Biotechnology, University of Wroclaw, Wroclaw, Poland; University of Alabama at Birmingham, United States of America

## Abstract

Extracellular fibroblast growth factor 1 (FGF1) acts through cell surface tyrosine kinase receptors, but FGF1 can also act directly in the cell nucleus, as a result of nuclear import of endogenously produced, non-secreted FGF1 or by transport of extracellular FGF1 via endosomes and cytosol into the nucleus. In the nucleus, FGF1 can be phosphorylated by protein kinase C δ (PKCδ), and this event induces nuclear export of FGF1. To identify intracellular targets of FGF1 we performed affinity pull-down assays and identified nucleolin, a nuclear multifunctional protein, as an interaction partner of FGF1. We confirmed a direct nucleolin-FGF1 interaction by surface plasmon resonance and identified residues of FGF1 involved in the binding to be located within the heparin binding site. To assess the biological role of the nucleolin-FGF1 interaction, we studied the intracellular trafficking of FGF1. In nucleolin depleted cells, exogenous FGF1 was endocytosed and translocated to the cytosol and nucleus, but FGF1 was not phosphorylated by PKCδ or exported from the nucleus. Using FGF1 mutants with reduced binding to nucleolin and a FGF1-phosphomimetic mutant, we showed that the nucleolin-FGF1 interaction is critical for the intranuclear phosphorylation of FGF1 by PKCδ and thereby the regulation of nuclear export of FGF1.

## Introduction

Fibroblast growth factor 1 (FGF1) belongs to the heparin binding fibroblast growth factor family, which consists of 22 members involved in a variety of cellular responses during embryonic development and in adult organisms. FGF1 regulates proliferation, differentiation, cell survival, and apoptosis [1]. FGF1-activity is usually mediated in a paracrine fashion by binding to and activation of high affinity, tyrosine kinase FGF receptors (FGFR1-4) on the cell surface. The activation of FGFRs leads to activation of downstream signaling cascades such as the PLCγ/PKC, PI3K/Akt, and Ras/MAP kinase pathways [2].

In addition to activation of FGFRs and their downstream signaling pathways, extracellular FGF1 can cross cellular membrane and translocate to the cytosol and nucleus [3], [4]. Also endogenously produced, non-secreted FGF1 can be found in the cell nucleus [5], [6]. Nuclear FGF1 has been implicated in DNA synthesis and proliferation [7], and it has been shown to play a role in cell differentiation, survival and in modulating p53-induced apoptosis [5], [6], [8]. In addition to FGF1, exogenous FGF2, epidermal growth factors (EGFs), cytokines, as well as receptors such as EGF receptors, FGFR1, and FGFR2, can be transported to the nucleus where they regulate cellular activities such as proliferation, survival and tumor progression [3], [4], [9]–[12].

The translocation of extracellular FGF1 into the cell is a regulated process and requires binding to cell surface FGFR1 or FGFR4 [13]–[15]. Also, the activity of several intracellular proteins such as PI3K [16] and p38 MAPK [17] is necessary for this process. Furthermore, it was shown that translocation of endocytosed FGF1 to the cytosol depends on a vesicular transmembrane electric potential indicating that FGF1 is translocated to the cytosol from an endosomal compartment [18]. The nuclear import of FGF1 is regulated by two nuclear localization sequences (NLS), one monopartite [19] and one bipartite [20]. Inside the nucleus, FGF1 is phosphorylated by PKCδ on serine 130 [21]. Exportin-1 binds phosphorylated FGF1, and FGF1 is then rapidly exported in a nuclear export sequence (NES)-mediated fashion to the cytosol where it is subsequently degraded [21], [22].

More studies on the mechanism of action of intracellular/nuclear FGF1 are necessary to elucidate the role of intracellular FGF1, and we have aimed at identifying intracellular binding partners of FGF1. Previously, we have shown that FGF1 interacts with several intracellular proteins including casein kinase 2 (CK2) [23], and FGF1 intracellular binding protein (FIBP) [24], a protein found to be crucial for FGF-dependent left-right asymmetry patterning in zebrafish [25]. Furthermore, FGF1 interacts with LRRC59/ribosome binding protein p34 [26], which is required for translocation of FGF1 from the cytosol to the nucleus [27]. FGF1 has also been found to interact with GRP75mortalin [28] and p53 [6]. We show here that FGF1, as well as FGF2, interacts with nucleolin, a multifunctional nucleolar protein involved in cellular processes such as growth, cell cycle regulation, transcription, apoptosis, ribosome biogenesis, and nucleocytoplasmic trafficking of ribosome particles [29] as well as other proteins [30]–[33]. It has previously been published that nuclear FGF2 interacts with and stimulates CK2, which leads to phosphorylation of nucleolin [34]. Here, we explore the role of the FGF1-nucleolin interaction in intracellular trafficking of FGF1 and demonstrate that nucleolin regulates phosphorylation of FGF1 by PKCδ in the nucleus and thereby regulates nuclear export of FGF1.

## Materials and Methods

### Antibodies and reagents

[^35^S]methionine, [^33^P]phosphate, and [γ-^33^P]ATP were obtained from Amersham Pharmacia Biotech. Recombinant FGF1 and *in vivo* transcribed [^35^S]methionine labelled FGF1 (^35^S-FGF1) were produced as described previously [16]. Leptomycin B (LMB) and thapsigargin were from Sigma-Aldrich. Rottlerin and bafilomycin A1 (BafA1) were from Calbiochem. The following primary antibodies were used with the catalogue numbers indicated in parentheses: mouse anti-nucleolin (anti-C23) (sc-8031), goat anti-FGF1 (sc-1884), goat anti-FGF2 (sc-74412), and rabbit anti-PKCδ (sc-937) from Santa Cruz Biotechnology, mouse anti-lamin A (ab8980) and mouse anti-GAPDH-HRP (ab9482) from Abcam, rabbit anti p44/42 MAPK (#9102) and rabbit anti-phospho-PKCδ (Thr 505) (#9374) from Cell Signalling Technology, mouse anti-Hsp90 (610419) from BD Transduction Laboratories, and mouse anti-γ-tubulin (T6557) from Sigma. Secondary antibodies conjugated to HRP were from Jackson Immune-Research Laboratories. Recombinant active PKCδ was from SignalChem and full length FGF1 was from Abcam. Heparin-Sepharose CL-6B affinity resin and Glutathione Sepharose 4 Fast Flow affinity resin was from GE Healthcare, and Ni-NTA Superflow was from Qiagen. Protease inhibitor cocktail tablets (EDTA-free, Complete) were from Roche Diagnostics and phosphatase inhibitor cocktails were from Sigma-Aldrich. Dynabeads M-280 Streptavidin was purchased from Invitrogen and anti-c-Myc antibody (Agarose) was from Abcam. 4-20% Precise Protein Gels were from Thermo Scientific and mini-PROTEAN TGX precast gels from Bio-Rad. Immobilon-P membranes were from Millipore and Trans-Blot Turbo 0.2 µm PVDF from Bio-Rad.

### Cell lines and bacterial strains

Human normal foreskin fibroblast cell line BJ, human embryonic kidney 293 (HEK 293) cell line and mouse fibroblast cell line NIH3T3 were from ATCC. Fibroblast cell lines were grown in Quantum 333 medium (PAA laboratories GmbH). The HEK 293 cell line and the previously described U2OSR1 cell line [35] were grown in Dulbeccòs modified Eaglès medium (DMEM, Gibco) supplemented with 10 % FBS (PAA Laboratories GmbH). Both the Quantum 333 medium and DMEM medium were supplemented with antibiotics (100 U/ml penicillin and 100 µg/ml streptomycin, from Gibco) and the cells were grown in a 5 % CO_2_ atmosphere at 37°C. The cells were seeded into tissue culture plates the day preceding the start of the experiments. *E. coli* strains Bl21(DE3)pLyS Rosetta and Bl21(DE3)pLyS were from Merck, and Bl21(DE3)-RIL was from Stratagene.

### Plasmids

The nucleolin coding sequence was obtained from GenScript. Sequences encoding FGF1 (Ala-residues 21-154) [36], FGF2 (residues 1-154) and a fragment of nucleolin (residues 284–707, nucleolin-C) [37] were cloned into a pDEST15 expression vector (with an N-terminal GST). The sequence encoding FGF1 was also cloned into a pET-SBP expression vector (with an N-terminal SBP), a pDEST17 expression vector (with an N-terminal hexahistidine peptide) and a pcDNA3 vector (with an N-terminal myc sequence).

### siRNAs and transfection

The nucleolin targeting siRNA was obtained from Qiagen (SI02654925). LRRC59 targeting siRNA was described previously [27]. Scrambled control siRNA was obtained from Thermo Scientific Dharmacon (CD-001810-01-20). For siRNA transfection studies, U2OSR1 cells (1x10^5^cells/ml) were seeded out and after 24 h the cells were transfected with 50 nM of the nucleolin targeting siRNA and control targeting siRNA, and 75 nM of the LRRC59 targeting siRNA using Lipofactamine RNAiMax Transfection Reagent (Invitrogen) according to the procedure given by the company. Seven hours after transfection, 10% FBS was added to the cells, and the cells were cultured for 72 h before further experiments.

Transient expression of myc-FGF1 was performed by transfecting HEK 293 cells with plasmid DNA using Escort IV Transfection Reagent (Sigma) in Minimum Essential Medium (MEM, Gibco) according to the manufacturer’s protocol. After 7 h the medium was changed for DMEM supplemented with 10 % FBS and the cells were grown for 24 h.

### Design of mutations

Potential interaction sites on the surface of the FGF1 molecule were determined using the bioinformatic web servers meta-PPISP [38], ConSurf [39] and SWAKK [40]. 18 surface mutations disturbing putative binding sites were selected and introduced using the Quick Change Site-Directed Mutagenesis protocol (Stratagene).

### Protein expression and purification

The C-terminal fragment of nucleolin (residues 284–707, nucleolin-C) was expressed as a fusion protein with an N-terminal GST in the *E. coli* Bl21(DE3)pLysS Rosetta strain. The protein was purified from the bacterial lysate using a Glutathione Sepharose 4 Fast Flow column, followed by rTEV protease cleavage and tandem GSH-Sepharose HiTrap and Heparin-Sepharose HiTrap chromatography using the Äkta Prime system (GE Healthcare). Purity and molecular mass were verified by SDS-PAGE and matrix-assisted laser desorption/ionization time-of-flight MS on an Applied Biosystems 4800 (Life Technologies).

FGF1 and FGF2 wild type proteins or mutants were produced with an N-terminal GST tag in *E. coli* Bl21(DE3)pLysS or Bl21(DE3)-RIL strains and then purified on a Glutathione Sepharose 4 Fast Flow column. To obtain tag-free proteins, fusion proteins were cleaved by rTEV protease and subjected to tandem GSH-Sepharose HiTrap and Heparin-Sepharose HiTrap chromatography using the Äkta Prime system (GE Healthcare). We obtained 17 soluble proteins among 18 FGF1 mutant constructs. His-FGF1 as well as SBP-FGF1 were produced in *E. coli* Bl21(DE3)pLysS or Bl21(DE3)-RIL strains and then purified on a Heparin-Sepharose CL-6B column. Protein homogeneity and identity were checked by SDS-PAGE and mass spectrometry (Applied Biosystems). To verify the native conformation of purified FGFs, circular dichroism (Jasco J-715 spectropolarimeter) and fluorescence (Jasco FP-750 or FP-8500 spectrofluorimeter) measurements were applied as described previously [36], [41]. FGF1 and FGF2 were biotinylated using EZ-Link Sulfo-NHS-LC-Biotin (Thermo Scientific Pierce) in a 1∶1 molar ratio for 5 min.

### Pull-down assays

BJ, NIH3T3, or transfected HEK 293 cells were lysed in lysis buffer (20 mM Tris-HCl pH 7.4, 150 mM NaCl, 1 mM EDTA, 1% Triton X-100 supplemented with a protease inhibitor cocktail) and sonicated for 3×10 s. Cellular debris was pelleted by centrifugation. Cleared HEK 293 lysate was incubated with 20 µl of anti-c-Myc antibody (Agarose) for 1 h at room temperature. Cleared BJ or NIH3T3 cell lysates were incubated with 73 pmol of recombinant GST-FGF1, GST-FGF2 or GST protein alone (negative control) for 1 h followed by incubation with 30 µl of Glutathione Sepharose 4 Fast Flow for 1 h at room temperature. Cell lysates were also incubated with recombinant SBP-FGF1, biotynylated FGF2 or His-FGF1 followed by incubation with 50 µl of Streptavidin-coated Dynabeads or 30 µl of Ni-NTA Superflow for 1 h. In all cases, the resins were washed four times in PBS with 1 % Triton X-100 before the protein complexes were eluted by 10 min boiling in SDS sample buffer. Protein complexes were subjected to SDS-PAGE and western blotting.

A similar procedure was applied to test for direct binding of recombinant nucleolin to FGF1 and FGF2. 73 pmol of FGFs were incubated with an equal molar amount of nucleolin.

### Mass spectrometry analysis

To identify proteins interacting with FGF1 we used recombinant SBP-FGF1 and cell lysate from NIH3T3 cells. 10 µg of SBP-FGF1 was incubated with 100 µl of Streptavidin-coated Dynabeads at 4°C. After 2 h the beads were washed three times with PBS with 1% Triton X-100 and incubated with lysed cells for 2 h at 4°C. Then, the beads were washed four times in PBS with 1% Triton X-100 before the protein complexes were eluted by 10 min boiling in SDS sample buffer. The proteins were analyzed by SDS–PAGE followed by Coomassie blue staining. In the control experiment cell lysate was incubated with Streptavidin-coated Dynabeads alone. Protein bands different from the control were cut from the gel, trypsinized and analyzed by MS. For selected samples MS/MS experiments were performed. Mass spectra were acquired on an Ultraflex II MALDI-TOF/TOF instrument from Bruker Daltonics (Bremen, Germany) controlled by FlexControl software (version 2.4, Bruker Daltonics) at the Core Facility for Proteomics and Mass Spectrometry at Oslo University Hospital-Rikshospitalet, Institute of Immunology, Oslo, Norway.

### Surface plasmon resonance

The interaction between nucleolin and FGF1 or FGF2 was investigated by surface plasmon resonance (SPR) analysis using a Biacore 3000 system (GE Healthcare). Recombinant nucleolin-C (10 µg/ml in 10 mM sodium acetate buffer, pH 4.0) was covalently immobilized on a carboxymethylated dextran sensor chip (CM4, GE Healthcare) using an amine coupling kit (GE Healthcare) on the level of ∼ 540 response units (RU). A reference flow cell was prepared by subjecting a surface to the amine coupling procedure in the absence of nucleolin. HEPES-buffered saline-P (10 mM HEPES, 0.15 M NaCl, 3 mM EDTA, 0.005% P20, pH 7.4) was used as immobilization running buffer. 60 µl aliquots of FGF1 or FGF2 in running buffer (PBS with 0.1 mg/ml BSA, 0.005 % P20, pH 7.3) were injected at 25°C at a flow rate of 30 µl/min and specific protein-protein interactions were measured. Dissociation of growth factors from nucleolin was monitored over a 4 min period. Binding of selected FGF1 variants to nucleolin was examined upon their injection at the concentration of 654 nM onto a CM5 sensor chip (GE Healthcare) with immobilized nucleolin (on the level of ∼ 5970 RU). Regeneration of the sensor chip surface was performed with 1.0 M NaCl. The data were analyzed using the BIAevaluation 3.1 software (GE Healthcare). Final sensorgrams were generated by subtracting the response in the reference flow cell from the responses in the sample flow cell. Interaction Maps and calculations of kinetic parameters for the interaction of the C-terminal fragment of nucleolin with FGF1 and FGF2 were made by Ridgeview Diagnostics AB, Uppsala, Sweden.

### Cell fractionation

Cells incubated with FGFs were washed in a high salt/low-pH-buffer (HSLP, 2 M NaCl, 20 mM sodium acetate, pH 4.0) to remove surface bound FGFs and HEPES medium before fractionation. For fractionation of cells into cytoplasmic and nuclear fractions cells were lysed in lysis buffer (0.15 M KCl, 40 mM Tris, pH 7.2, 1 % Triton X-100, 2 mM EDTA) supplemented with protease and phosphatase inhibitor cocktails. The Triton X-100 soluble fraction was collected as the cytoplasmic fraction and the insoluble fraction obtained by centrifugation of lysates was collected as the nuclear fraction. The nuclear fraction was washed in lysis buffer and sonicated. For fractionation of cells into membrane, cytosolic and nuclear fractions we used a digitonin fractionation method, described previously [21]. Cells incubated with FGFs for 6 or 10 h (in addition to inhibitors LMB (5 ng/ml), thapsigargin (1 µg/ml), rottlerin (10 µM) or BafA1 (10 nM) were washed with HSLP-buffer to remove surface bound FGFs. The cells were permeabilized with 20 µg/ml digitonin in PBS and incubated at 25°C for 5 min and on ice for additional 30 min to allow the cytosol to diffuse into the buffer. The buffer was collected and denoted the cytosolic fraction. The remainder of the cells was lysed in lysis buffer, and the Triton X-100 soluble fraction was designated the membrane fraction. The insoluble material was sonicated and designated the nuclear fraction. FGFs were extracted from the subcellular fractions by adsorption to Heparin-Sepharose beads, washed and then eluted and analyzed by SDS-PAGE and fluorography and/or immunoblotting. In addition, samples of the fractions were loaded directly onto SDS-PAGE for analysis by immunoblotting.

### In vivo phosphorylation of FGF1

The method was previously described in [16], [27]. Briefly, U2OSR1 cells were starved for 24 h and the cellular ATP pool was radiolabelled by incubation with 25 µCi/ml [^33^P]phosphate in phosphate free medium overnight. Then the cells were stimulated with 100 ng/ml recombinant FGF1 and 10 U/ml heparin for 6 h. All inhibitors/drugs were added at the same time as FGF1 and were present during the entire incubation. After 6 h the cells were washed with a HSLP-buffer and HEPES medium containing heparin to remove surface bound FGF1. The cells were then lysed and sonicated (for analysis of total cell lysate) or fractionated into cytoplasmic (containing both cytosol and membranes) and nuclear fractions. The cell lysate/subcellular fractions were incubated with Heparin-Sepharose beads to bind FGF1 and the beads were washed extensively. FGF1 is highly resistant to trypsin when bound to heparin, and the beads (with bound FGF1) were treated with 2 µg/ml TPCK- treated trypsin (Sigma-Aldrich) to remove most proteins other than FGF1. FGF1 was eluted from the beads in an SDS-buffer and subjected to SDS-PAGE and western blotting. The blots were exposed to fluorography to detect [^33^P]-phosphorylated FGF1 (^33^P-FGF1). ^33^P-FGF1 represents the fraction of internalized FGF1 that was translocated to the cytosol/nucleus since the specific phosphorylation of FGF1 by PKCδ can only occur in the cytosolic and nuclear compartment due to the intracellular localization of PKCδ. The total amount of internalized FGF1 (which includes material in endosomes) was detected on the same blot by anti-FGF1 and immunoblotting. For analysis of proteins other than FGF1, an aliquot of each lysate/fraction was withdrawn before binding to Heparin-Sepharose and analyzed separately by SDS-PAGE and immunoblotting.

### In vitro phosphorylation of FGF1 by PKCδ

Recombinant wild type or mutant FGF1 (200 ng) was incubated with recombinant active PKCδ (50 ng) for 30 min at 30°C in a buffer containing 40 µCi [γ-^33^P]ATP, 25 mM MOPS pH 7.2, 12.5 mM β-glycerol-phosphate, 25 mM MgCl_2_, 5 mM EGTA, 2 mM EDTA, 0.25 mM DTT, and 50 µM unlabelled ATP. The FGFs were thereafter adsorbed to Heparin-Sepharose beads, washed, and separated by SDS-PAGE and analyzed by fluorography and anti-FGF1 by immunoblotting.

## Results

### FGF1 and FGF2 interacts with nucleolin

To identify intracellular interaction partners for FGF1, affinity pull-down assays using cell lysates and recombinant FGF1 tagged with streptavidin binding peptide (SBP-FGF1) as a bait were performed. FGF1-interacting proteins were separated by SDS-PAGE (Figure S1), then extracted, trypsinized and analyzed by mass spectrometry (MS). The most frequently identified protein was nucleolin. The identity of nucleolin was confirmed by MS/MS experiments. Other proteins found using this approach were lamin A, nucleophosmin, lamin A/C, annexin A2, Ap-2 complex subunit alpha-2, carbamoyl-phosphate synthase, and major vault protein.

**Figure 1:**
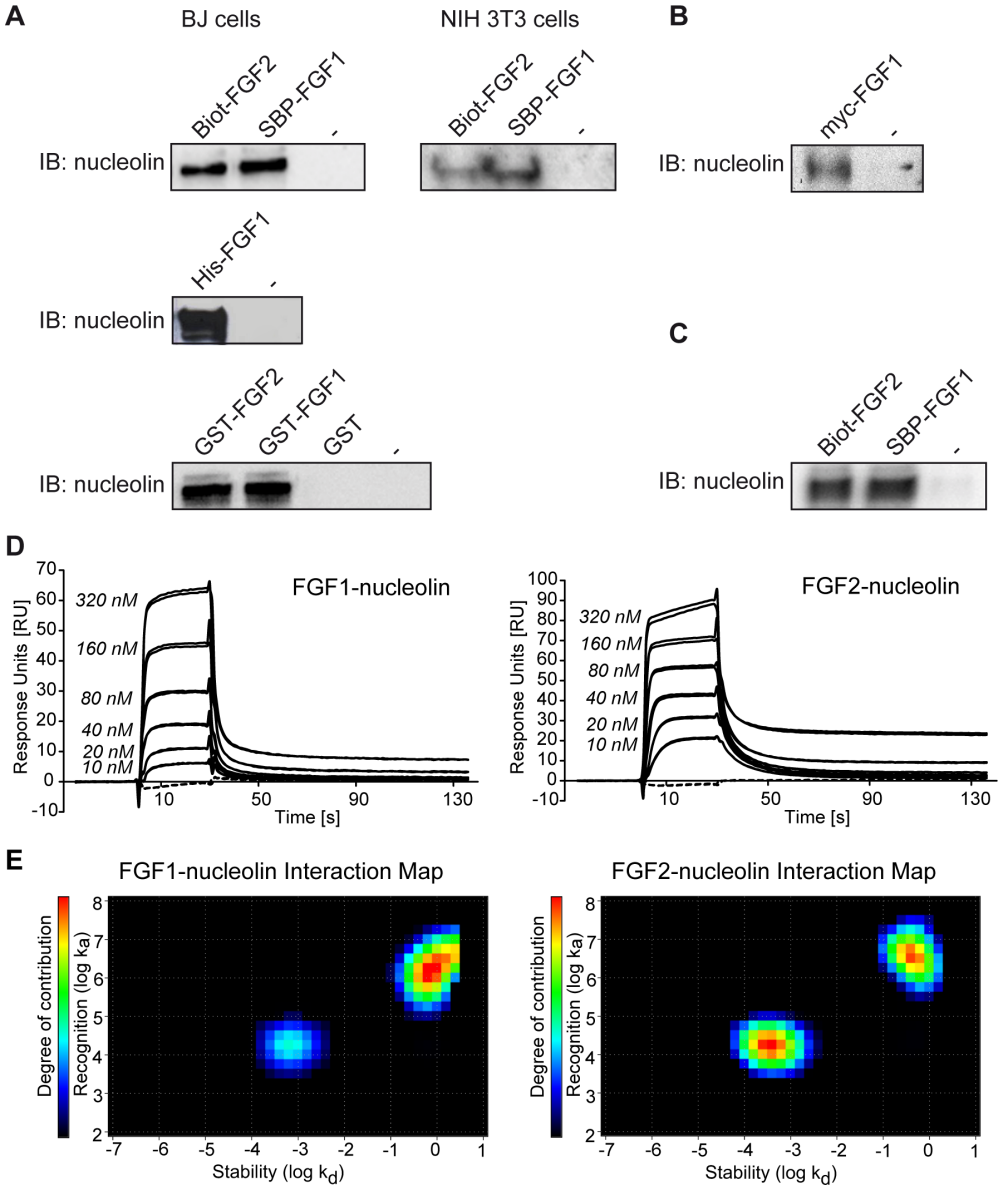
Nucleolin binds to FGF1 and FGF2. A) Proteins from cleared cell lysates (BJ or NIH3T3 cells) were pulled down by SBP-FGF1, Biot-FGF2, His-FGF1, GST-FGF1, GST-FGF2, GST protein, or no protein (–), and respective resins (Streptavidin-coated Dynabeads, Ni-NTA Superflow or Glutathione Sepharose™ 4 Fast Flow). Protein complexes were subjected to SDS-PAGE and immunoblotting (IB) with an anti-nucleolin antibody. B) HEK 293 cells transiently transfected with myc-FGF1 were lysed and then subjected to immunoprecipitation followed by SDS-PAGE and immunoblotting (IB) with an anti-nucleolin antibody. Non-transfected cells were used as controls. C) Recombinant nucleolin-C was incubated with recombinant SBP-FGF1 or Biot-FGF2 and Streptavidin-coated Dynabeads, then washed and analyzed by IB with an anti-nucleolin antibody. D) SPR analysis was performed using nucleolin-C immobilized on a CM4 sensor chip at the level of ∼540 RU and recombinant FGF1 or FGF2 were injected as analyte on the chip at increasing concentrations (10 nM to 320 nM) in duplicates (solid lines). Running buffer was injected in duplicates as a control (dashed lines). E) Interaction Maps from kinetic SPR data obtained for FGF1 and FGF2 binding to nucleolin-C. The contribution of a particular k_a_ or k_d_ in the overall binding event is shown by colors, from black (no contribution) to red (strong contribution).

The interaction between FGF1 and nucleolin was verified by *in vitro* binding experiments followed by immunoblotting using a nucleolin-specific antibody. To ensure the independence of the results from experimental settings we applied three constructs of FGF1 fused to different tags (SBP-FGF1, hexahistidine-tagged FGF1 (His-FGF1) and glutathione S-transferase-tagged FGF1 (GST-FGF1)), three different types of resins (Streptavidin-coated Dynabeads, Glutathione Sepharose or Ni-NTA-Agarose) for growth factor immobilization, and lysates from two different cell lines (BJ and NIH3T3) as a source of cellular proteins. We also used biotinylated FGF2 (Biot-FGF2) and glutathione S-transferase-tagged FGF2 (GST-FGF2) to investigate whether nucleolin binding is specific for FGF1. As shown in Figure 1A, nucleolin was present in the fraction pulled down from the cell lysates by each of the FGF1 or FGF2 constructs. As a negative control we used either beads alone or beads incubated with GST. These experiments show that FGF1 or FGF2 can form a complex with nucleolin. To provide more physiological conditions, we also tested whether FGF1 could interact with endogenous nucleolin in mammalian cells. Similarly to previously shown pull-down experiments with recombinant FGFs, we were able to pull-down nucleolin from HEK 293 cells by precipitating transiently overexpressed myc-FGF1 using myc-agarose (Figure 1B). Nucleolin was not detected in untransfected cells upon incubation with myc-agarose. This result confirms that nucleolin is an interaction partner of FGF1 in mammalian cells.

To test if the interaction between FGF1 or FGF2 and nucleolin is direct, we performed *in vitro* binding assays using recombinant Biot-FGF2 or SBP-FGF1 and a recombinant C-terminal fragment (residues 284–707) of nucleolin (nucleolin-C). In agreement with a study of Yang *et al*., we were not able to express the full-length nucleolin since its N-terminal domain is very acidic and hinders the solubility of the recombinant protein [35]. We found that FGF1 and FGF2, but not beads alone, bound to nucleolin-C, indicating that there is a direct binding between these proteins (Figure 1C). Next, to quantify the strength of the interactions, we applied the SPR technique using the Biacore system. Concentration-dependent responses for FGF1 and FGF2 (concentration range 10–320 nM) upon binding to immobilized nucleolin-C confirmed an *in vitro* interaction of these proteins (Figure 1D). Despite the monomeric state of FGFs, association and dissociation curves did not fit well to the simple 1∶1 Langmuir binding model and thus exhibited a complexity in the interactions. In order to assess the binding parameters the method called Interaction Map was applied [42], which allows for the resolution of the contributing interactions without knowledge about kinetic constants or degree of heterogeneity (K. Andersson, and M. Malmqvist, Method for the analysis of solid biological objects, patent application WO 2010033069, 2008). Interaction Map calculations confirmed the complexity of the interaction between growth factors and nucleolin-C (Figure 1E). In the case of FGF1-nucleolin binding, analysis of the sensorgrams suggested the existence of two parallel binding processes, one of apparent affinity K_D_ = 4.0×10^−8^ M (k_a_ = 1.8×10^4^ M^−1^s^−1^ k_d_ = 7.0×10^−4^ s^−1^) and one of approximate affinity 4.8×10^−7^ M (k_a_ = 1.7×10^6^ M^−1^s^−1^ k_d_ = 8.3×10^−1^ s^−1^). The weaker interaction seems to be approximately three times as abundant as the stronger interaction (Table S1). The FGF2-nucleolin sensorgrams also indicated the contribution of two components in the overall binding, one of approximate affinity K_D_ = 2.2×10^−8^ M (k_a_ = 1.7×10^4^ M^−1^s^−1^ k_d_ = 3.8×^−4^ s^−1^) and one of approximate affinity 1.3×10^−7^ M (k_a_ = 3.5×10^6^ M^−1^s^−1^ k_d_ = 4.4×10^−1^ s^−1^). The two interactions contributed equally to the detected signal (Table S1).

In experiments with recombinant FGF1 we used its truncated form (Ala-FGF1^21−154^), which is commonly used in FGF1 research and well characterized [43]. There was no difference in the proliferative response of cells to full length FGF1 and its truncated form (Figure S2A). We also compared the ability of both FGF1 forms to bind to nucleolin and found no difference (Figure S2B).

### Identification of residues of FGF1 involved in nucleolin binding

**Figure 2:**
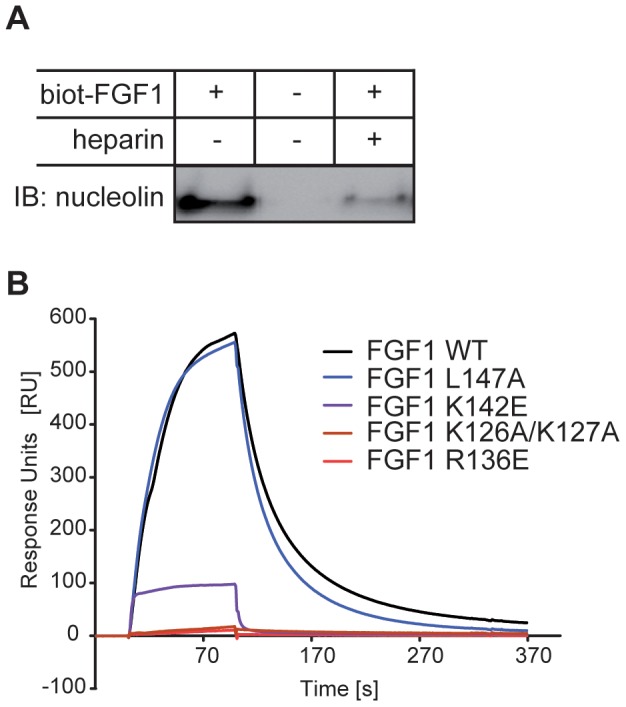
The positively charged heparin-binding site of FGF1 is responsible for binding to nucleolin. A) Biot-FGF1 was incubated with recombinant nucleolin-C and Streptavidin-coated Dynabeads in the presence or absence of heparin, then washed and analyzed by SDS-PAGE and immunoblotting with an anti-nucleolin antibody. B) SPR analysis of the binding of FGF1 mutants (K126A/K127A, R136E, K142E and L147A) to nucleolin-C immobilized on a CM5 sensor chip at the level of ∼5790 RU. FGF1 wild-type or its variants were injected as analytes at a concentration of 654 nM.

An *in vitro* binding assay with recombinant nucleolin-C and biotinylated FGF1 (Biot-FGF1) was performed in the presence of heparin, a negatively charged polyanion that binds to positively charged amino acid residues on the surface of FGF1. As shown in Figure 2A and Figure S3, the presence of heparin remarkably diminished the nucleolin-C interaction with FGF1 suggesting that the residues involved in nucleolin binding are localized within the heparin-binding region of the FGF1 molecule.

To identify more precisely residues of FGF1 responsible for the interaction with nucleolin, we constructed and tested 17 mutants with disturbance in putative binding sites on the surface of the FGF1 molecule (Figure S4A). SPR experiments using FGF1 mutants at concentration of 654 nM showed a significant decrease in the binding response for three variants: K126A/K127A, R136E and K142E (Figure S4B, Figure 2 B). For the K126A/K127A and the K142E variants we observed significantly weaker binding to nucleolin than for the wild-type, whereas for the R136E mutant we could not detect any response even at 1 µM concentration. All of these amino acid substitutions are located within the positively charged heparin-binding site of FGF1, suggesting an electrostatic nature of the FGF1 binding to nucleolin. As a control we used an L147A variant with a substitution outside the heparin binding region. Figure S5 shows elution profiles of selected mutants from a Heparin-Sepharose column with a linear NaCl gradient.

### Nucleolin is not involved in nuclear import of exogenous FGF1

Endocytosed FGF1 can translocate to the cytosol by crossing endosomal membranes and thereafter be imported into the nucleus [18]. Nucleolin has been shown to play a role in nucleocytoplasmic trafficking of proteins and ribosomal subunits [29]–[33], [44], and we therefore investigated if nucleolin is involved in any of the intracellular transport steps of externally added FGF1.

**Figure 3:**
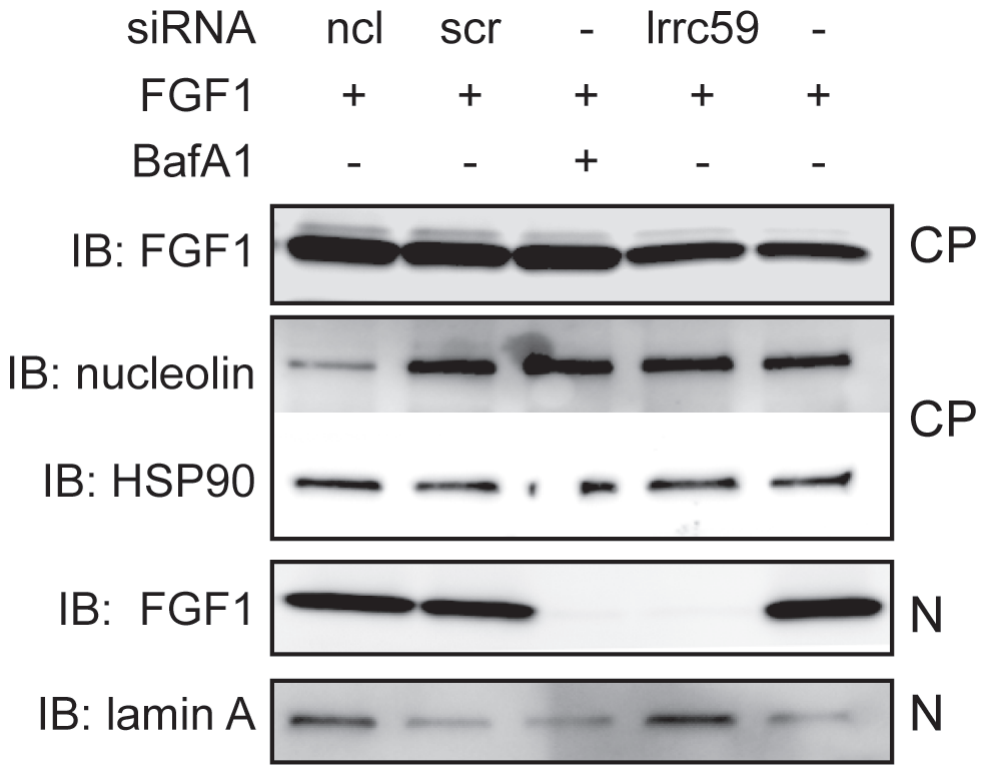
Nucleolin is not involved in nuclear import of exogenous FGF1. U2OSR1 cells were transfected with siRNA against nucleolin (ncl) or LRRC59 (lrrc59) or scrambled (scr) RNA or left non-transfected (–). The cells were serum starved for 24 h and incubated with 100 ng/ml FGF1 and 10 U/ml heparin, and in one case also 10 nM BafA1, for 6h. The cells were washed, lysed, and fractionated into a cytoplasmic fraction (CP) and a nuclear fraction (N). FGF1 was extracted from both fractions by adsorption to Heparin-Sepharose and analyzed by SDS-PAGE and immunoblotting (IB) with anti-FGF1. The cellular fractions were also analyzed for nucleolin, HSP90, and lamin A by IB as indicated.

To study the nuclear import of FGF1, we used U2OS cells stably transfected with FGFR1 (U2OSR1) that were depleted for nucleolin by transfection with nucleolin-specific siRNA. Since nucleolin is an essential protein, the siRNA parameters were chosen so as to give partial nucleolin depletion to ensure cell viability. The cells were serum starved for 24 h before stimulation with FGF1 for 6 h to allow endocytosis and entry into the nucleus [15]. Then, the cells were washed to remove surface bound FGF1, lysed and fractionated into a cytoplasmic fraction, which included membranes and endosomes, and a nuclear fraction. The fractions were analyzed for FGF1 by immunoblotting. In scrambled siRNA treated cells as well as in nucleolin depleted cells, FGF1 was detected in both the nuclear and the cytoplasmic fractions (Figure 3). In accordance with previous reports, nuclear entry of FGF1 was efficiently inhibited by BafA1, which inhibits vacuolar proton pumps and thereby represses translocation of FGF1 from endosomes to the cytosol [18], and by depletion of the LRRC59 protein thereby inhibiting transport of FGF1 from the cytosol to the nucleus [27]. BafA1 treatment and LRRC59 depletion were used here and in later experiments as negative controls for FGF1 translocation into the cytosol/nucleus. Our data indicates that nucleolin is not involved in the endocytic uptake or nuclear translocation of exogenous FGF1.

### Nucleolin is required for intracellular phosphorylation of FGF1

**Figure 4:**
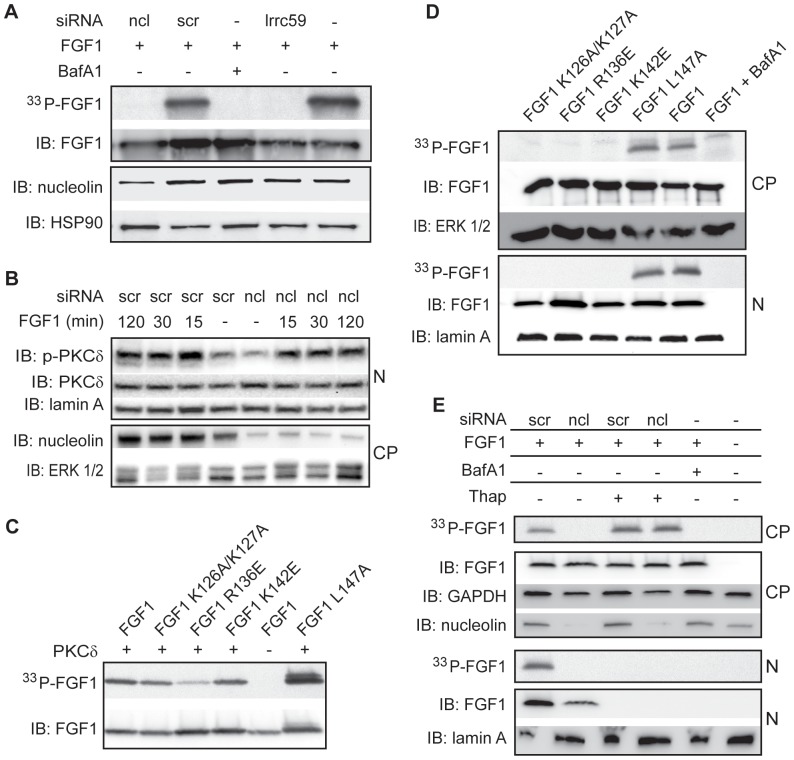
Nucleolin is required for intracellular phosphorylation of FGF1. A) U2OSR1 cells were transfected with siRNA as indicated, serum starved for 24 h and labelled by [^33^P]phosphate, and thereafter stimulated with 100 ng/ml unlabelled, recombinant FGF1 in the presence of 10 U/ml heparin, and 10 nM BafA1 where indicated, for 6 h. FGF1 was extracted from total cell lysates by adsorption to Heparin-Sepharose and analyzed by SDS PAGE and fluorography to detect *in vivo* phosphorylation, and immunoblotting (IB) to detect FGF1. The lysates were also analyzed for nucleolin and HSP90 by IB. B) U2OSR1 cells transfected with siRNA as indicated were serum starved for 24 h, stimulated with FGF1 and heparin for the indicated time and then lysed and fractionated into a cytoplasmic (CP) and a nuclear (N) fraction before analysis by SDS-PAGE and IB for phospho-PKCδ and total PKCδ. The fractions were also analyzed for nucleolin, lamin A, and ERK1/2 by IB. C) Recombinant FGF1 or FGF1 mutants as indicated, were incubated with recombinant, active PKCδ and [γ-^33^P]ATP and thereafter analyzed for *in vitro* phosphorylation by fluorography, and IB to show loading. D) U2OSR1 cells were serum starved and labelled with [^33^P]phosphate and stimulated with FGF1 or FGF1 mutants as indicated for 6 h. The cells were fractionated into cytoplasmic and nuclear fractions. FGFs were extracted from the fractions by binding to Heparin-Sepharose and analyzed by fluorography to detect *in vivo* phosphorylation and immunoblotting (IB) to detect FGFs. Fractions were also analyzed for marker proteins by IB as indicated. E) U2OSR1 cells were transfected with siRNA as indicated, serum starved for 24 h and labelled with [^33^P]phosphate and stimulated with FGF1 in the absence or presence of 1 µg/ml thapsigargin. The cells were fractionated and analyzed as described in (D).

Translocated FGF1 can be phosphorylated by PKCδ on serine 130. This phosphorylation event has been shown to occur in the nucleus and to regulate the availability of a NES and thereby nuclear export of FGF1 [22]. To study if nucleolin is involved in nuclear phosphorylation of FGF1, U2OSR1 cells depleted for nucleolin were serum starved, treated with [^33^P]phosphate to label the cellular ATP pool, and then incubated with unlabelled recombinant FGF1 for 6 h. Total cell lysates were analyzed for intracellularly [^33^P]-phosphorylated FGF1 (^33^P-FGF1). As shown in Figure 4A, ^33^P-FGF1 was detected in control cells (scrambled or no siRNA treated), however, for nucleolin depleted cells, as well as for LRRC59 depleted or BafA1 treated cells, no phosphorylated FGF1 was observed. This result was confirmed by siRNA against nucleolin obtained from two different companies with non-overlapping sequences (Figure S6). This result suggests that nucleolin is involved in regulating intracellular FGF1 phosphorylation. We also confirmed our results using full length FGF1. Nucleolin depletion inhibited intracellular phosphorylation of the long as well as the short form of FGF1 (Figure S7), and this experiment also demonstrated that the long form of FGF1 was converted into a shorter form before translocation into the nucleus.

We analyzed if nucleolin depletion affected the activity of PKCδ, the kinase responsible for FGF1 phosphorylation. Cells were stimulated with FGF1 and analyzed for activated (Thr505 phosphorylated) PKCδ by immunoblotting. As shown in Figure 4B, activation of nuclear PKCδ appeared to be similar in nucleolin depleted and scrambled siRNA treated cells. Thus, nucleolin does not seem to influence the presence or activation of PKCδ in the nucleus.

To further test the role of nucleolin in the phosphorylation of FGF1, we studied phosphorylation of three FGF1 mutants with highly reduced ability to interact with nucleolin (FGF1 K126A/K127A, R136E and K142E, as described above) and one control mutant with no disturbance in the FGF1-nucleolin interaction (FGF1 L147A). All of these FGF1 mutants could be phosphorylated by PKCδ in an *in vitro* assay (Figure 4C). However, within U2OSR1 cells, no phosphorylation of the mutants, except the control variant, could be detected, although their nuclear import was comparable to that of wild type FGF1 (Figure 4D). This result suggests that an interaction between nucleolin and FGF1 is necessary for intracellular phosphorylation of FGF1 by PKCδ.

We have previously observed that when cells are treated with thapsigargin, a drug that can be used to inhibit nuclear import of proteins, translocated FGF1 is phosphorylated in the cytosol, due to inhibited nuclear import of FGF1 as well as PKCδ [21], [27]. We therefore analyzed if FGF1 could be phosphorylated in the cytosol in nucleolin depleted cells in the presence of thapsigargin. As shown in Figure 4E, nucleolin depletion abolished phosphorylation of FGF1, while with the concomitant treatment with thapsigargin phosphorylated FGF1 could be detected in the cytoplasmic fraction. This finding corroborates our previous result that the activity of PKCδ is unaffected by nucleolin depletion while it also indicates that the regulatory role of nucleolin in the process of PKCδ mediated phosphorylation of FGF1 is restricted to a nuclear localization.

To test if nucleolin functions as an enhancer for the PKCδ mediated phosphorylation of FGF1, we studied the *in vitro* phosphorylation of FGF1 by PKCδ in the presence or absence of recombinant nucleolin, but we were unable to detect a stimulatory effect of nucleolin in phosphorylation of FGF1 (Figure S8). The role of nucleolin in PKCδ mediated phosphorylation of FGF1 thus appears to be specific for an intracellular and nuclear location.

### Nucleolin regulates nuclear export of FGF1

**Figure 5:**
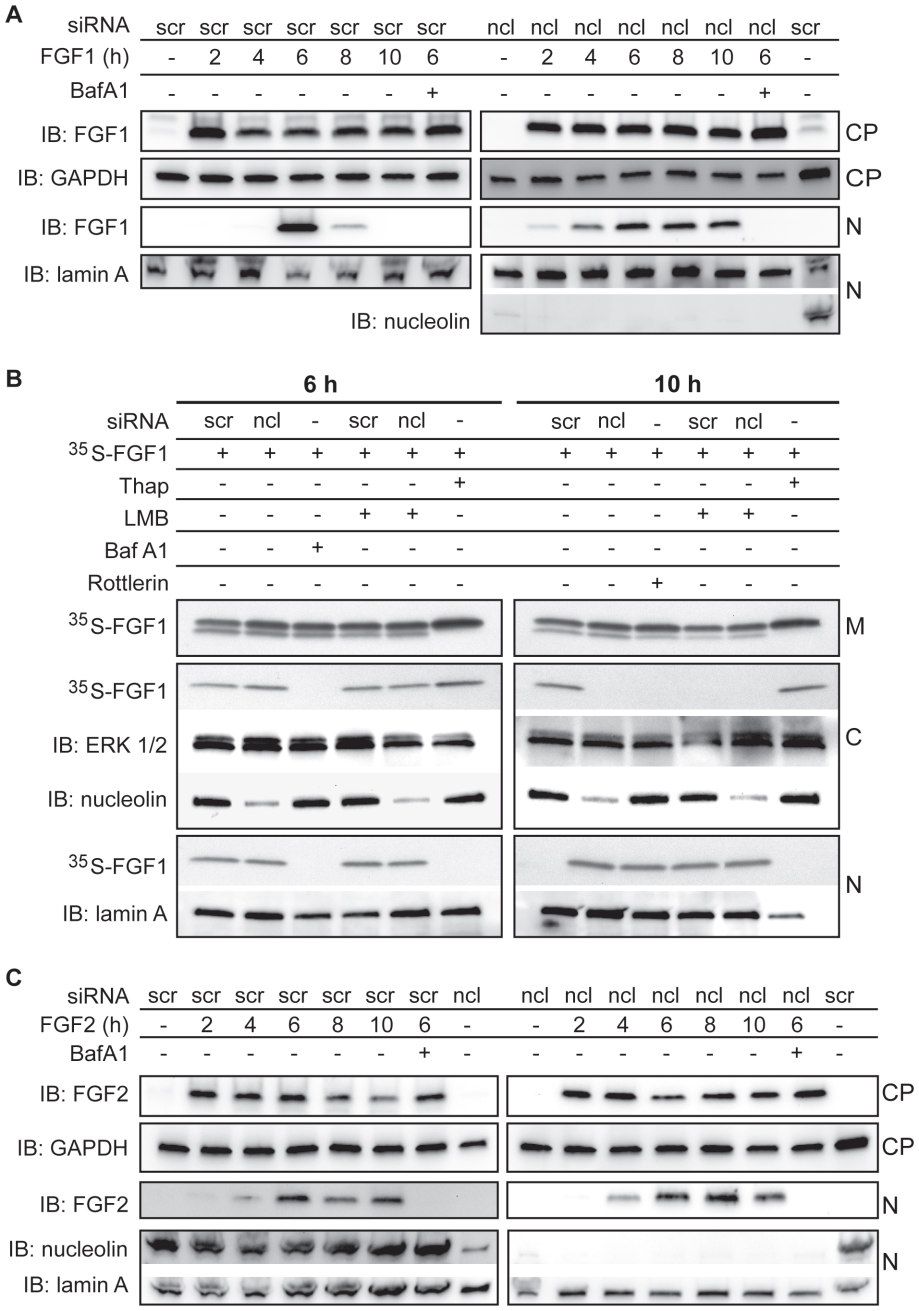
Nucleolin is required for nuclear export of FGF1, but does not influence nuclear localization of FGF2. A) U2OSR1 cells were transfected with siRNA as indicated, serum starved for 24 h, and incubated with 100 ng/ml FGF1 and 10 U/ml heparin for 2, 4, 6, 8 and 10 h, and also 10 nM BafA1 where indicated. The cells were lysed and fractionated into a cytoplasmic fraction (CP) and a nuclear fraction (N), and FGF1 was extracted from the fractions by adsorption to Heparin-Sepharose and analyzed by SDS-PAGE and immunoblotting (IB) with anti-FGF1. The cellular fractions were also analyzed for nucleolin, GAPDH, and lamin A by IB as indicated. B) U2OSR1 cells were transfected with siRNA as indicated, and serum starved for 24 h before stimulation with ^35^S-FGF1 for 6 h (left panel) or 10 h (right panel) in the absence or presence of 10 nM BafA1, 5 ng/ml LMB, 10 µM rottlerin or 1µg/ml thapsigargin, as indicated. The cells were fractionated into membrane (M), cytosolic (C) and nuclear (N) fractions. ^35^S-FGF1 was extracted from all fractions by adsorption to Heparin-Sepharose and analyzed by SDS-PAGE and fluorography. The fractions were also analyzed for nucleolin, ERK1/2 and lamin A by IB. C) The experiment was performed as in (A) except the cells were stimulated with 100 ng/ml FGF2 instead of FGF1.

Since nucleolin depletion abolished *in vivo* phosphorylation of FGF1, and phosphorylation regulates nuclear export of FGF1 [21], we investigated the nuclear export of FGF1 in nucleolin depleted cells. Previous studies have demonstrated that the amount of FGF1 in the nucleus reaches a peak about 6 h after the addition of FGF1 to the cell medium, and FGF1 is thereafter exported from the nucleus and degraded in the cytosol [21]. Scrambled siRNA treated and nucleolin depleted cells were incubated with FGF1 for 2-10 h and then the nuclear fractions were analyzed for FGF1. Figure 5A shows that a peak amount of nuclear FGF1 was observed at 6 h in scrambled treated cells, while the amount of nuclear FGF1 was reduced after 8 h and undetectable after 10 h, which is in accordance with previously published data [21]. In nucleolin depleted cells, the amount of nuclear FGF1 did not decline notably between 6 h and 10 h after FGF1 addition, suggesting that nucleolin depletion inhibits nuclear export and thereby prolongs the localization of translocated FGF1 in the nucleus (Figure 5A).

To study in more detail the intracellular localization of FGF1in nucleolin depleted cells we investigated the amount of FGF1 in membrane, cytosolic and nuclear fractions. Cells were treated with *in vitro* [^35^S]methionine-labelled FGF1 (^35^S-FGF1) for 6 or 10 h (Figure 5B). In scrambled treated cells and nucleolin depleted cells, FGF1 was found in the nucleus, as well as in the cytosol, after 6 h. In scrambled treated cells, no FGF1 was detected in the nucleus after 10 h, but some was still found in the cytosol, presumably due to the efficient export from the nucleus. In nucleolin depleted cells, on the other hand, FGF1 was located in the nuclear fraction and not in the cytosolic fraction after 10 h, indicating that nucleolin depletion inhibited FGF1 nuclear export. A similar effect was observed when the nuclear export was blocked by LMB, a specific inhibitor of exportin-1, or when the phosphorylation of FGF1 was inhibited by rottlerin, an inhibitor of PKCδ. When nuclear import was blocked by thapsigargin, translocated FGF1 was detected only in the cytosolic fraction (6 h and 10 h). These data indicate that nucleolin is a crucial factor for regulation of nuclear export of FGF1.

Similarily to FGF1, exogenous FGF2 can translocate into the nucleus [11], however, FGF2 does not contain a PKCδ phosphorylation site and the mechanisms for its nuclear export is unknown. Since nucleolin also interacts with FGF2, we investigated if nucleolin plays a role in nuclear export of FGF2 as observed for FGF1. As can be seen in Figure 5C, the amount of nuclear FGF2 after incubation for 2–10 h is the same in nucleolin depleted cells as in scrambled treated cells. This indicates that nucleolin does not influence nuclear shuttling of FGF2 within the time frame studied.

### Nucleolin regulates nuclear export of FGF1 via phosphorylation

**Figure 6:**
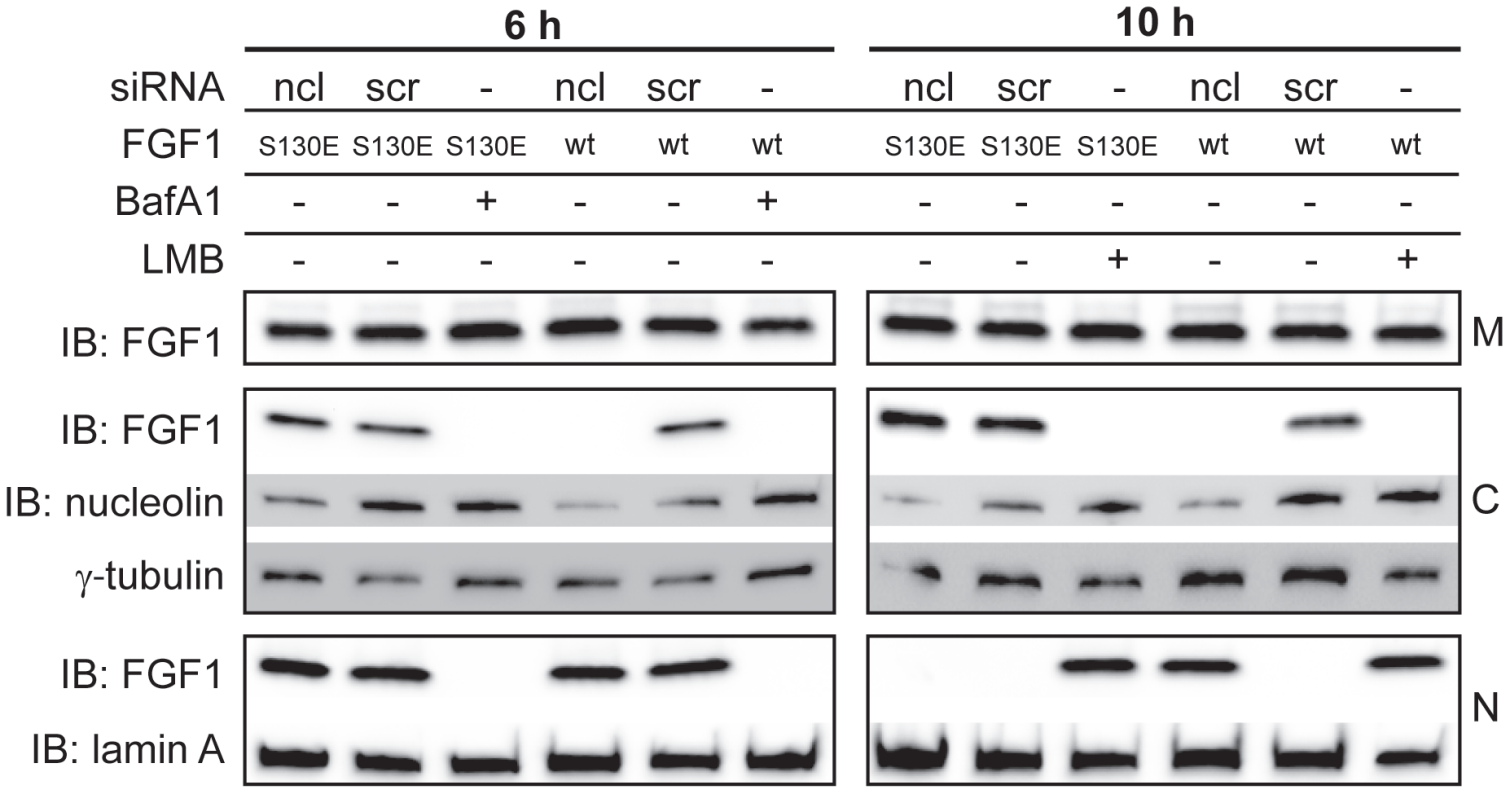
Nucleolin regulates nuclear export of FGF1 via phosphorylation. U2OSR1 cells were transfected with siRNA as indicated, and serum starved for 24 h before stimulation for 6 h (left panel) or 10 h (right panel) with 100 ng/ml FGF1 (wt) or 100 ng/ml FGF1 S130E and 10 U/ml heparin. Cells were also treated with 10 nM BafA1 or 5 ng/ml LMB, where indicated. The cells were fractionated into membrane (M), cytosolic (C) and nuclear (N) fractions, and FGFs were extracted from the fractions by adsorption to Heparin-Sepharose and analyzed by SDS-PAGE and immunoblotting (IB). The fractions were also analyzed for nucleolin, γ-tubulin and lamin A by IB, as indicated.

To determine if nucleolin played a role in nuclear export of FGF1 by regulating its phosphorylation only, or if nucleolin was involved directly in nuclear trafficking, we studied the nuclear export of FGF1 S130E, a mutant which mimics FGF1 phosphorylated on serine 130 [45]. Cells depleted for nucleolin were stimulated with FGF1 S130E or wild type FGF1 for 6 h or 10 h and then fractionated into membrane, cytosolic, and nuclear fractions (Figure 6). Wild type FGF1 was found in the nuclear fraction and not in the cytosolic fraction after 6 h as well as 10 h in nucleolin depleted cells. However, the FGF1 S130E mutant was present in the cytosolic fraction and not in the nuclear fraction after 10 h, both in scrambled and nucleolin depleted cells, indicating that it was efficiently exported from the nucleus. This result indicates that nucleolin is not directly involved in the nuclear export of FGF1, but rather regulates FGF1 nuclear export via regulating its phosphorylation by PKCδ.

## Discussion

In this study we show that FGF1 interacts with the nuclear protein nucleolin, and that nucleolin regulates nucleocytoplasmic trafficking of FGF1. We found that nucleolin is required for phosphorylation of FGF1 on serine 130 by PKCδ in the nucleus. This phosphorylation is a key regulatory event for nuclear export of FGF1 and nucleolin is thus a crucial regulator of FGF1 nuclear export.

The ability of exogenous FGF1 to translocate into the nucleus was discovered more than two decades ago [19], [46], while the mechanism for translocation and the intracellular action of FGF1 are continuously being investigated [12]. To elucidate the role of nuclear FGF1 we aimed to identify intracellular targets for FGF1 by mass spectrometry-based proteomic studies. We identified nucleolin as an interaction partner of FGF1 and confirmed the direct *in vitro* binding between FGF1, and also FGF2, and nucleolin by SPR technique. The SPR kinetic studies displayed a complex interaction suggesting two parallel binding processes between FGF1/FGF2 and nucleolin. The K_D_ was estimated to in the range from 4.0×10^−8^ M to 4.8×10^−7^ M for FGF1 and in the range from 2.2×10^−8^ M to 1.3×10^−7^ M for FGF2. FGF2 has previously been suggested to interact with nucleolin [34], [47], but the direct FGF2-nucleolin and FGF1-nucleolin interactions are for the first time presented here.

Bioinformatic analysis enabled us to obtain 17 FGF1 variants with mutated putative interaction sites. SPR analysis revealed that most of them did not exhibit any significant change in nucleolin binding. However, four amino acid residues were found to be involved in nucleolin binding, K126, K127, R136 and K142. These are located at the heparin-binding site, indicating a dual function of the positively charged patch on the FGF1 molecule. This suggests that the heparin-binding sites in FGF1 responsible for heparin interaction outside the cell are engaged again after translocation of FGF1 into the nucleus, then constituting a binding site for the intracellular protein nucleolin. As FGF1 is a relatively small protein, our finding points to a great economy of the FGF1 structure. Interestingly, nucleolin has previously been shown to bind to the heparin-binding site on endostatin which leads to internalization of endostatin and stimulation of antiangiogenic activities [48].

Nucleolin is a multifunctional protein implicated in a variety of cellular processes including ribosome biogenesis, proliferation, and differentiation [29], [44], [49]. Also, nucleolin has been found to regulate nucleocytoplasmic shuttling of several proteins including endostatin [33], lactoferrin [32], transforming growth factor β receptor [30], and the US11 protein of Herpes Simplex Virus 1 [31], as well as ribosomal proteins and subunits [29]. We therefore studied the uptake and intracellular trafficking of exogenous FGF1 in nucleolin depleted cells. We found that the intranuclear phosphorylation of FGF1 was abolished and that the nuclear export of FGF1 was severely impaired. Nucleolin is an essential protein in the cell and inhibition or depletion of nucleolin has previously been shown to lead to cell cycle arrest, defects in centrosome duplication and nucleolar disruption [50]. Therefore, as suggested before, we studied the role of nucleolin in cells only partially depleted for nucleolin [51]. These partially depleted cells appeared viable, and although the effect on FGF1 phosphorylation was severe, the nucleolin depletion had no apparent effect on the endocytic uptake of FGF1 or the translocation of FGF1 from endosomes into the cytosol and nucleus. Nucleolin depletion had also no detectable effect on the activity or the nuclear import of PKCδ, suggesting that nucleolin rather has a direct role in facilitating PKCδ-mediated phosphorylation of FGF1. One possibility is that FGF1 requires binding to nucleolin in order to be a substrate for PKCδ. To verify if this was the case, we tested FGF1 mutants that had a reduced or abolished binding to nucleolin (*in vitro*) and found that they were not phosphorylated intracellularly, although their nuclear import was comparable to that of wild type FGF1, and they could be phosphorylated by PKCδ *in vitro*. Furthermore, we found that wild type FGF1 could be phosphorylated in nucleolin depleted cells when the cells were treated with thapsigargin, a drug that inhibits nuclear import of FGF1 as well as PKCδ and thereby allows for PKCδ-FGF1 interaction in the cytosol. We therefore hypothesize that nucleolin stabilizes the interaction between FGF1 and PKCδ, and that this action is specific for a nuclear location. Possibly, nucleolin acts as a scaffolding protein and may induce a conformational change in the FGF1 molecule, allowing S130 of FGF1 to be exposed and available for the action of PKCδ.

Phosphorylation of FGF1 alters its conformation leading to exposure of a functional leucine-rich type NES at the C-terminus, and thereby regulates nuclear export of FGF1 [22]. We found that in nucleolin depleted cells, FGF1 remained in the nucleus several hours longer than in control cells, indicating a requirement of nucleolin for nuclear export of FGF1. By stimulating nucleolin depleted cells with an FGF1 mutant which mimics FGF1 phosphorylated on serine 130 (FGF1 S130E), we could distinguish if nucleolin was only involved in phosphorylation of FGF1 or if it was involved directly in the nuclear export process. We found that nucleolin depletion did not significantly inhibit nuclear export of FGF1 S130E, indicating that nucleolin controls FGF1 nuclear export mainly *via* regulating its phosphorylation by PKCδ. Although nucleolin was found to interact with FGF2 as well as FGF1, we did not observe any change in the nucleocytoplasmic trafficking of exogenous FGF2 upon nucleolin depletion. Unlike FGF1, translocated FGF2 binds to the protein translokin, which is important for nuclear import of FGF2 [52], [53]. In the nucleus FGF2 can bind and stimulate CK2, which induces phosphorylation of nucleolin [34]. Nuclear FGF2 can also interact with the transcription factor Upstream Binding Factor (UBF), and directly regulate rRNA transcription [47]. Possibly, the lack of nucleolin mediated trafficking of FGF2 is due to the lack of a PKCδ phosphorylation site on FGF2.

Nucleolin consists of three functional domains, an N-terminal domain with several acidic stretches, a C-terminal domain rich in glycine/arginine, and a central domain containing two to four RNA recognition motifs [49]. Further, nucleolin goes through several post translational modifications which targets nucleolin to different cellular compartments where it carries out different functions [29]. We showed here that FGF1 interacts with a fragment of nucleolin consisting of residues 284–707, which constitutes the C-terminal domain and the four RNA binding motifs [37]. Nucleolin has been shown to interact with several other proteins including ErbB4 receptor [54], Hdm2 [55], ribosomal protein L26 [56], and p53 [57], reflecting its many functions in the cell. The interaction between FGF1 and nucleolin suggests that FGF1 could regulate the functions of nucleolin or *vice versa*. Interestingly, both nucleolin and nuclear FGF1 have been implicated in regulation of p53. Nucleolin is involved in translational regulation of p53 mRNA [58], and stabilization of the p53-protein [55], [59], while nuclear FGF1 protects cells from p53-regulated apoptosis [5], [6]. Therefore it is possible that the interaction of FGF1 with nucleolin is involved in the anti-apoptotic function of translocated FGF1. FGF1 mutants defective in nucleolin binding are not phosphorylated and exported from the nucleus, and therefore may act longer by a potential nuclear pathway to protect the cells from apoptosis. More studies are necessary to clarify the biological role of FGF1 in the nucleus and how the roles of FGF1 and nucleolin intersect. Nevertheless, nuclear export of FGF1 is probably important in the regulation of its intracellular activity and the data presented here clearly implicate nucleolin as a regulator of the nuclear export of FGF1.

## Supporting Information

Figure S1 **Coomassie stained SDS-PAGE gel of FGF1-interacting proteins.**
(DOCX)

Figure S2 **Comparison of truncated form of FGF1 and full length FGF1.**
(DOCX)

Figure S3 **SPR shows that heparin prevents FGF1 binding to nucleolin.**
(DOCX)

Figure S4 **Mutational analysis of FGF1 binding to nucleolin.**
(DOCX)

Figure S5 **Elution profiles of FGF1.**
(DOCX)

Figure S6 **Non-overlapping siRNA sequences against nucleolin inhibits phosphorylation of FGF1 by PKCδ.**
(DOCX)

Figure S7 **Nucleolin is required for intracellular phosphorylation of full lenght and truncated forms of FGF1.**
(DOCX)

Figure S8 **Nucleolin does not influence phosphorylation of FGF1 by PKCδ** ***in vitro*** **.**
(DOCX)

Table S1 **Kinetic parameters of FGF1 and FGF2 binding to recombinant nucleolin-C.**
(DOCX)

## References

[1] Ornitz DM, Itoh N (2001) Fibroblast growth factors. Genome Biol 2: REVIEWS3005.1–REVIEWS3005.12. 11276432 10.1186/gb-2001-2-3-reviews3005 PMC138918

[2] Mohammadi M, Olsen SK, Ibrahimi OA (2005) Structural basis for fibroblast growth factor receptor activation. Cytokine Growth Factor Rev 16: 107–137. 15863029 10.1016/j.cytogfr.2005.01.008

[3] Olsnes S, Klingenberg O, Wiedlocha A (2003) Transport of exogenous growth factors and cytokines to the cytosol and to the nucleus. Physiol Rev 83: 163–182. 12506129 10.1152/physrev.00021.2002

[4] Planque N (2006) Nuclear trafficking of secreted factors and cell-surface receptors: new pathways to regulate cell proliferation and differentiation, and involvement in cancers. Cell Commun Signal 4: 7. 17049074 10.1186/1478-811X-4-7 PMC1626074

[5] Bouleau S, Grimal H, Rincheval V, Godefroy N, Mignotte B, et al (2005) FGF1 inhibits p53-dependent apoptosis and cell cycle arrest via an intracrine pathway. Oncogene 24: 7839–7849. 16091747 10.1038/sj.onc.1208932

[6] Rodriguez-Enfedaque A, Bouleau S, Laurent M, Courtois Y, Mignotte B, et al (2009) FGF1 nuclear translocation is required for both its neurotrophic activity and its p53-dependent apoptosis protection. Biochim Biophys Acta 1793: 1719–1727. 19765618 10.1016/j.bbamcr.2009.09.010

[7] Wiedlocha A, Falnes PO, Madshus IH, Sandvig K, Olsnes S (1994) Dual mode of signal transduction by externally added acidic fibroblast growth factor. Cell 76: 1039–1051. 7511061 10.1016/0092-8674(94)90381-6

[8] Renaud F, Desset S, Oliver L, Gimenez-Gallego G, Van OE, et al (1996) The neurotrophic activity of fibroblast growth factor 1 (FGF1) depends on endogenous FGF1 expression and is independent of the mitogen-activated protein kinase cascade pathway. J Biol Chem 271: 2801–2811. 8576258 10.1074/jbc.271.5.2801

[9] Brand TM, Iida M, Li C, Wheeler DL (2011) The nuclear epidermal growth factor receptor signaling network and its role in cancer. Discov Med 12: 419–432. 22127113 PMC3305885

[10] Jans DA, Hassan G (1998) Nuclear targeting by growth factors, cytokines, and their receptors: a role in signaling? Bioessays 20: 400–411. 9670813 10.1002/(SICI)1521-1878(199805)20:5<400::AID-BIES7>3.0.CO;2-R

[11] Sorensen V, Nilsen T, Wiedlocha A (2006) Functional diversity of FGF-2 isoforms by intracellular sorting. Bioessays 28: 504–514. 16615083 10.1002/bies.20405

[12] Wiedlocha A, Sorensen V (2004) Signaling, internalization, and intracellular activity of fibroblast growth factor. Curr Top Microbiol Immunol 286: 45–79. 15645710 10.1007/978-3-540-69494-6_3

[13] Klingenberg O, Wiedlocha A, Rapak A, Khnykin D, Citores L, et al (2000) Requirement for C-terminal end of fibroblast growth factor receptor 4 in translocation of acidic fibroblast growth factor to cytosol and nucleus. J Cell Sci 113 ( Pt 10): 1827–1838. 10.1242/jcs.113.10.1827 10769213

[14] Sorensen V, Wiedlocha A, Haugsten EM, Khnykin D, Wesche J, et al (2006) Different abilities of the four FGFRs to mediate FGF-1 translocation are linked to differences in the receptor C-terminal tail. J Cell Sci 119: 4332–4341. 17003104 10.1242/jcs.03209

[15] Zakrzewska M, Sorensen V, Jin Y, Wiedlocha A, Olsnes S (2011) Translocation of exogenous FGF1 into cytosol and nucleus is a periodic event independent of receptor kinase activity. Exp Cell Res 317: 1005–1015. 21223966 10.1016/j.yexcr.2011.01.003

[16] Klingenberg O, Wiedocha A, Citores L, Olsnes S (2000) Requirement of phosphatidylinositol 3-kinase activity for translocation of exogenous aFGF to the cytosol and nucleus. J Biol Chem 275: 11972–11980. 10766827 10.1074/jbc.275.16.11972

[17] Sorensen V, Zhen Y, Zakrzewska M, Haugsten EM, Walchli S, et al (2008) Phosphorylation of fibroblast growth factor (FGF) receptor 1 at Ser777 by p38 mitogen-activated protein kinase regulates translocation of exogenous FGF1 to the cytosol and nucleus. Mol Cell Biol 28: 4129–4141. 18411303 10.1128/MCB.02117-07 PMC2423127

[18] Malecki J, Wiedlocha A, Wesche J, Olsnes S (2002) Vesicle transmembrane potential is required for translocation to the cytosol of externally added FGF-1. EMBO J 21: 4480–4490. 12198150 10.1093/emboj/cdf472 PMC126202

[19] Imamura T, Engleka K, Zhan X, Tokita Y, Forough R, et al (1990) Recovery of mitogenic activity of a growth factor mutant with a nuclear translocation sequence. Science 249: 1567–1570. 1699274 10.1126/science.1699274

[20] Wesche J, Malecki J, Wiedlocha A, Ehsani M, Marcinkowska E, et al (2005) Two nuclear localization signals required for transport from the cytosol to the nucleus of externally added FGF-1 translocated into cells. Biochemistry 44: 6071–6080. 15835896 10.1021/bi047403m

[21] Wiedlocha A, Nilsen T, Wesche J, Sorensen V, Malecki J, et al (2005) Phosphorylation-regulated nucleocytoplasmic trafficking of internalized fibroblast growth factor-1. Mol Biol Cell 16: 794–810. 15574884 10.1091/mbc.E04-05-0389 PMC545912

[22] Nilsen T, Rosendal KR, Sorensen V, Wesche J, Olsnes S, et al (2007) A nuclear export sequence located on a beta-strand in fibroblast growth factor-1. J Biol Chem 282: 26245–26256. 17616529 10.1074/jbc.M611234200

[23] Skjerpen CS, Nilsen T, Wesche J, Olsnes S (2002) Binding of FGF-1 variants to protein kinase CK2 correlates with mitogenicity. EMBO J 21: 4058–4069. 12145206 10.1093/emboj/cdf402 PMC126148

[24] Kolpakova E, Wiedlocha A, Stenmark H, Klingenberg O, Falnes PO, et al (1998) Cloning of an intracellular protein that binds selectively to mitogenic acidic fibroblast growth factor. Biochem J 336 ( Pt 1): 213–222. 10.1042/bj3360213 PMC1219860 9806903

[25] Hong SK, Dawid IB (2009) FGF-dependent left-right asymmetry patterning in zebrafish is mediated by Ier2 and Fibp1. Proc Natl Acad Sci U S A 106: 2230–2235. 19164561 10.1073/pnas.0812880106 PMC2650137

[26] Skjerpen CS, Wesche J, Olsnes S (2002) Identification of ribosome-binding protein p34 as an intracellular protein that binds acidic fibroblast growth factor. J Biol Chem 277: 23864–23871. 11964394 10.1074/jbc.M112193200

[27] Zhen Y, Sorensen V, Skjerpen CS, Haugsten EM, Jin Y, et al (2012) Nuclear import of exogenous FGF1 requires the ER-protein LRRC59 and the importins Kpnalpha1 and Kpnbeta1. Traffic 13: 650–664. 22321063 10.1111/j.1600-0854.2012.01341.x

[28] Mizukoshi E, Suzuki M, Loupatov A, Uruno T, Hayashi H, et al (1999) Fibroblast growth factor-1 interacts with the glucose-regulated protein GRP75/mortalin. Biochem J 343 Pt 2: 461–466. PMC1220575 10510314

[29] Tajrishi MM, Tuteja R, Tuteja N (2011) Nucleolin: The most abundant multifunctional phosphoprotein of nucleolus. Commun Integr Biol 4: 267–275. 21980556 10.4161/cib.4.3.14884 PMC3187884

[30] Chandra M, Zang S, Li H, Zimmerman LJ, Champer J, et al (2012) Nuclear translocation of type I transforming growth factor beta receptor confers a novel function in RNA processing. Mol Cell Biol 32: 2183–2195. 22473997 10.1128/MCB.00320-12 PMC3372271

[31] Greco A, Arata L, Soler E, Gaume X, Coute Y, et al (2012) Nucleolin interacts with US11 protein of herpes simplex virus 1 and is involved in its trafficking. J Virol 86: 1449–1457. 22130536 10.1128/JVI.06194-11 PMC3264372

[32] Legrand D, Vigie K, Said EA, Elass E, Masson M, et al (2004) Surface nucleolin participates in both the binding and endocytosis of lactoferrin in target cells. Eur J Biochem 271: 303–317. 14717698 10.1046/j.1432-1033.2003.03929.x

[33] Song N, Ding Y, Zhuo W, He T, Fu Z, et al (2012) The nuclear translocation of endostatin is mediated by its receptor nucleolin in endothelial cells. Angiogenesis 15: 697–711. 22711211 10.1007/s10456-012-9284-y

[34] Bonnet H, Filhol O, Truchet I, Brethenou P, Cochet C, et al (1996) Fibroblast growth factor-2 binds to the regulatory beta subunit of CK2 and directly stimulates CK2 activity toward nucleolin. J Biol Chem 271: 24781–24787. 8798749 10.1074/jbc.271.40.24781

[35] Haugsten EM, Malecki J, Bjorklund SM, Olsnes S, Wesche J (2008) Ubiquitination of fibroblast growth factor receptor 1 is required for its intracellular sorting but not for its endocytosis. Mol Biol Cell 19: 3390–3403. 18480409 10.1091/mbc.E07-12-1219 PMC2488279

[36] Zakrzewska M, Krowarsch D, Wiedlocha A, Olsnes S, Otlewski J (2005) Highly stable mutants of human fibroblast growth factor-1 exhibit prolonged biological action. J Mol Biol 352: 860–875. 16126225 10.1016/j.jmb.2005.07.066

[37] Yang C, Kim MS, Chakravarty D, Indig FE, Carrier F (2009) Nucleolin Binds to the Proliferating Cell Nuclear Antigen and Inhibits Nucleotide Excision Repair. Mol Cell Pharmacol 1: 130–137. 20336191 10.4255/mcpharmacol.09.17 PMC2844761

[38] Qin S, Zhou HX (2007) meta-PPISP: a meta web server for protein-protein interaction site prediction. Bioinformatics 23: 3386–3387. 17895276 10.1093/bioinformatics/btm434

[39] Ashkenazy H, Erez E, Martz E, Pupko T, Ben-Tal N (2010) ConSurf 2010: calculating evolutionary conservation in sequence and structure of proteins and nucleic acids. Nucleic Acids Res 38: W529–W533. 20478830 10.1093/nar/gkq399 PMC2896094

[40] Liang H, Zhou W, Landweber LF (2006) SWAKK: a web server for detecting positive selection in proteins using a sliding window substitution rate analysis. Nucleic Acids Res 34: W382–W384. 16845032 10.1093/nar/gkl272 PMC1538794

[41] Zakrzewska M, Wiedlocha A, Szlachcic A, Krowarsch D, Otlewski J, et al (2009) Increased protein stability of FGF1 can compensate for its reduced affinity for heparin. J Biol Chem 284: 25388–25403. 19574212 10.1074/jbc.M109.001289 PMC2757240

[42] Svitel J, Balbo A, Mariuzza RA, Gonzales NR, Schuck P (2003) Combined affinity and rate constant distributions of ligand populations from experimental surface binding kinetics and equilibria. Biophys J 84: 4062–4077. 12770910 10.1016/S0006-3495(03)75132-7 PMC1302986

[43] Burgess WH (1991) Structure-function studies of acidic fibroblast growth factor. Ann N Y Acad Sci 638: 89–97. 1723865 10.1111/j.1749-6632.1991.tb49020.x

[44] Srivastava M, Pollard HB (1999) Molecular dissection of nucleolin's role in growth and cell proliferation: new insights. FASEB J 13: 1911–1922. 10544174

[45] Klingenberg O, Wiedlocha A, Olsnes S (1999) Effects of mutations of a phosphorylation site in an exposed loop in acidic fibroblast growth factor. J Biol Chem 274: 18081–18086. 10364261 10.1074/jbc.274.25.18081

[46] Imamura T, Tokita Y, Mitsui Y (1992) Identification of a heparin-binding growth factor-1 nuclear translocation sequence by deletion mutation analysis. J Biol Chem 267: 5676–5679. 1372009

[47] Sheng Z, Liang Y, Lin CY, Comai L, Chirico WJ (2005) Direct regulation of rRNA transcription by fibroblast growth factor 2. Mol Cell Biol 25: 9419–9426. 16227592 10.1128/MCB.25.21.9419-9426.2005 PMC1265826

[48] Fu Y, Chen Y, Luo X, Liang Y, Shi H, et al (2009) The heparin binding motif of endostatin mediates its interaction with receptor nucleolin. Biochemistry 48: 11655–11663. 19877579 10.1021/bi901265z

[49] Ginisty H, Sicard H, Roger B, Bouvet P (1999) Structure and functions of nucleolin. J Cell Sci 112 ( Pt 6): 761–772. 10.1242/jcs.112.6.761 10036227

[50] Ugrinova I, Monier K, Ivaldi C, Thiry M, Storck S, et al (2007) Inactivation of nucleolin leads to nucleolar disruption, cell cycle arrest and defects in centrosome duplication. BMC Mol Biol 8: 66. 17692122 10.1186/1471-2199-8-66 PMC1976620

[51] Chen J, Guo K, Kastan MB (2012) Interactions of nucleolin and ribosomal protein L26 (RPL26) in translational control of human p53 mRNA. J Biol Chem 287: 16467–16476. 22433872 10.1074/jbc.M112.349274 PMC3351294

[52] Bossard C, Laurell H, Van den BL, Meunier S, Zanibellato C, et al (2003) Translokin is an intracellular mediator of FGF-2 trafficking. Nat Cell Biol 5: 433–439. 12717444 10.1038/ncb979

[53] Meunier S, Navarro MG, Bossard C, Laurell H, Touriol C, et al (2009) Pivotal role of translokin/CEP57 in the unconventional secretion versus nuclear translocation of FGF2. Traffic 10: 1765–1772. 19804566 10.1111/j.1600-0854.2009.00985.x

[54] Di SA, Farin K, Pinkas-Kramarski R (2008) Identification of nucleolin as new ErbB receptors- interacting protein. PLoS One 3: e2310. 18523588 10.1371/journal.pone.0002310 PMC2390759

[55] Saxena A, Rorie CJ, Dimitrova D, Daniely Y, Borowiec JA (2006) Nucleolin inhibits Hdm2 by multiple pathways leading to p53 stabilization. Oncogene 25: 7274–7288. 16751805 10.1038/sj.onc.1209714

[56] Takagi M, Absalon MJ, McLure KG, Kastan MB (2005) Regulation of p53 translation and induction after DNA damage by ribosomal protein L26 and nucleolin. Cell 123: 49–63. 16213212 10.1016/j.cell.2005.07.034

[57] Daniely Y, Dimitrova DD, Borowiec JA (2002) Stress-dependent nucleolin mobilization mediated by p53-nucleolin complex formation. Mol Cell Biol 22: 6014–6022. 12138209 10.1128/MCB.22.16.6014-6022.2002 PMC133981

[58] Chen J, Guo K, Kastan MB (2012) Interactions of nucleolin and ribosomal protein L26 (RPL26) in the translational control of human p53 mRNA. J Biol Chem 287: 16467–16476. 22433872 10.1074/jbc.M112.349274 PMC3351294

[59] Bhatt P, d'Avout C, Kane NS, Borowiec JA, Saxena A (2012) Specific domains of nucleolin interact with Hdm2 and antagonize Hdm2-mediated p53 ubiquitination. FEBS J 279: 370–383. 22103682 10.1111/j.1742-4658.2011.08430.x PMC3262062

