# Supplementary material for: Nucleolin Regulates Phosphorylation and Nuclear Export of Fibroblast Growth Factor 1 (FGF1)
Source: PLoS One. 2014 Mar 4;9(3):e90687. doi: 10.1371/journal.pone.0090687 (PMC3942467; doi:10.1371/journal.pone.0090687)
Supplement: Figure S1 — Coomassie stained SDS-PAGE gel of FGF1-interacting proteins. (DOCX) [file pone.0090687.s001.docx]

**Figure S1.**

**
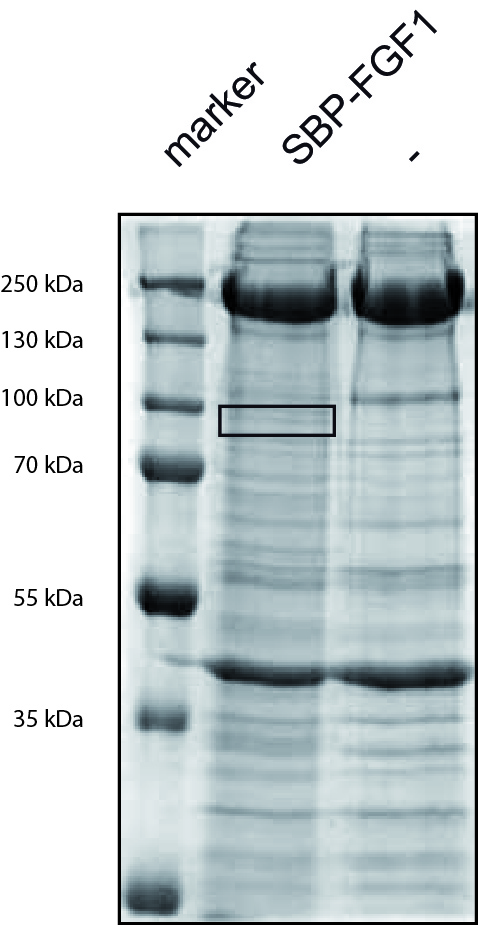
**

**Figure S1.** Coomassie stained SDS-PAGE gel of FGF1-interacting proteins isolated from affinity pull-down assay using NIH3T3 cell lysate and recombinant FGF1 tagged with streptavidin binding peptide (SBP-FGF1) as a bait. As a negative control NIH3T3 cell lysate was incubated with Streptavidin-coated Dynabeads. Nucleolin was identified by MS analysis in the gel fragment marked by rectangle box.
