# Supplementary material for: Nucleolin Regulates Phosphorylation and Nuclear Export of Fibroblast Growth Factor 1 (FGF1)
Source: PLoS One. 2014 Mar 4;9(3):e90687. doi: 10.1371/journal.pone.0090687 (PMC3942467; doi:10.1371/journal.pone.0090687)
Supplement: Figure S2 — Comparison of truncated form of FGF1 and full length FGF1. (DOCX) [file pone.0090687.s002.docx]

**Figure S2.**

**
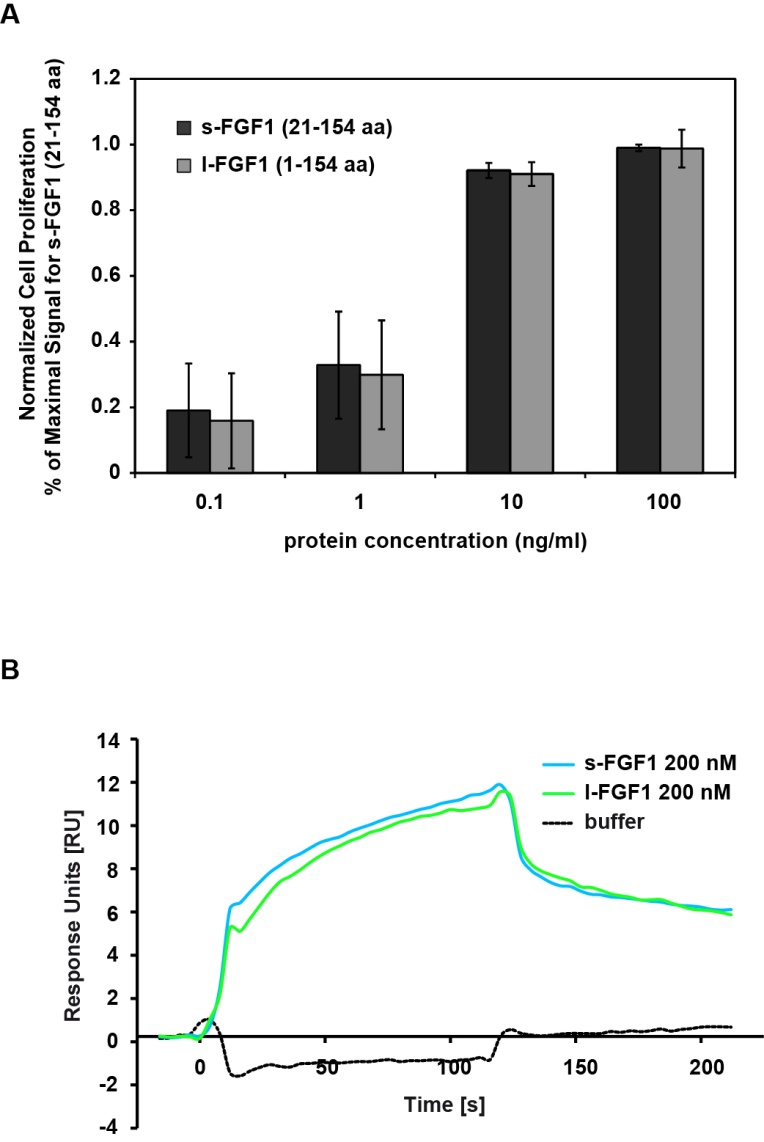
**

**Figure S2. Comparison of truncated form of FGF1 (short, 21-154) and full length FGF1 (long, 1-154).** (A) FGF1-induced cell proliferation of NIH 3T3 cells. AlamarBlue reagent was added to serum-starved cells that had been stimulated for 48 h with indicated concentrations of FGF1 (truncated (amino acids Ala-21-154) and full length (amino acids 1-154) in the presence of heparin (10 U/ml). The fluorescence corresponding to the number of cells was measured using EnVision multimode plate reader (PerkinElmer). The graph represents the mean ± SEM of four independent experiments. (B) FGF1 binding to nucleolin. SPR analysis was performed using nucleolin-C immobilized on a CM4 sensor chip at the level of ~540 RU and recombinant FGF1 was injected as analyte on the chip at 200 nM concentration. Running buffer was injected as a control.
