# Supplementary material for: Nucleolin Regulates Phosphorylation and Nuclear Export of Fibroblast Growth Factor 1 (FGF1)
Source: PLoS One. 2014 Mar 4;9(3):e90687. doi: 10.1371/journal.pone.0090687 (PMC3942467; doi:10.1371/journal.pone.0090687)
Supplement: Figure S3 — SPR shows that heparin prevents FGF1 binding to nucleolin. (DOCX) [file pone.0090687.s003.docx]

**Figure S3.**

**
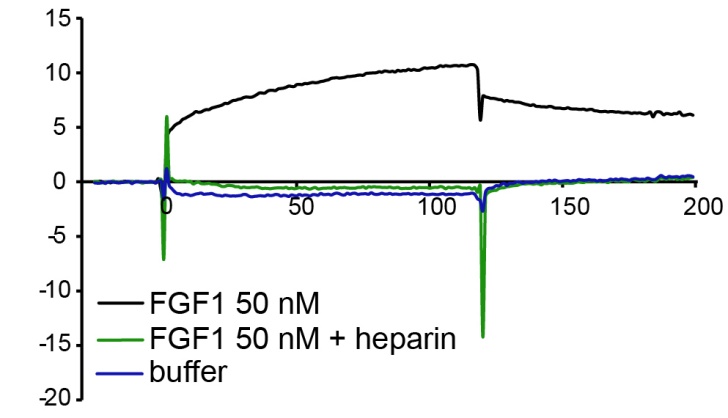
**

**Figure S3. SPR shows that heparin prevents FGF1 binding to nucleolin.** Nucleolin-C was immobilized on a CM4 chip at the level of ~ 540 RU. FGF1 was injected as an analyte at 50 nM concentration, in the absence or presence of heparin at a concentration of 76.4 U/ml. The applied heparin concentration was calculated to keep the heparin:FGF1 ratio of 10 U heparin per 100 ng FGF1, as used in the experiments performed on cells.
