# Supplementary material for: Nucleolin Regulates Phosphorylation and Nuclear Export of Fibroblast Growth Factor 1 (FGF1)
Source: PLoS One. 2014 Mar 4;9(3):e90687. doi: 10.1371/journal.pone.0090687 (PMC3942467; doi:10.1371/journal.pone.0090687)
Supplement: Figure S4 — Mutational analysis of FGF1 binding to nucleolin. (DOCX) [file pone.0090687.s004.docx]

**Figure S4.**

**
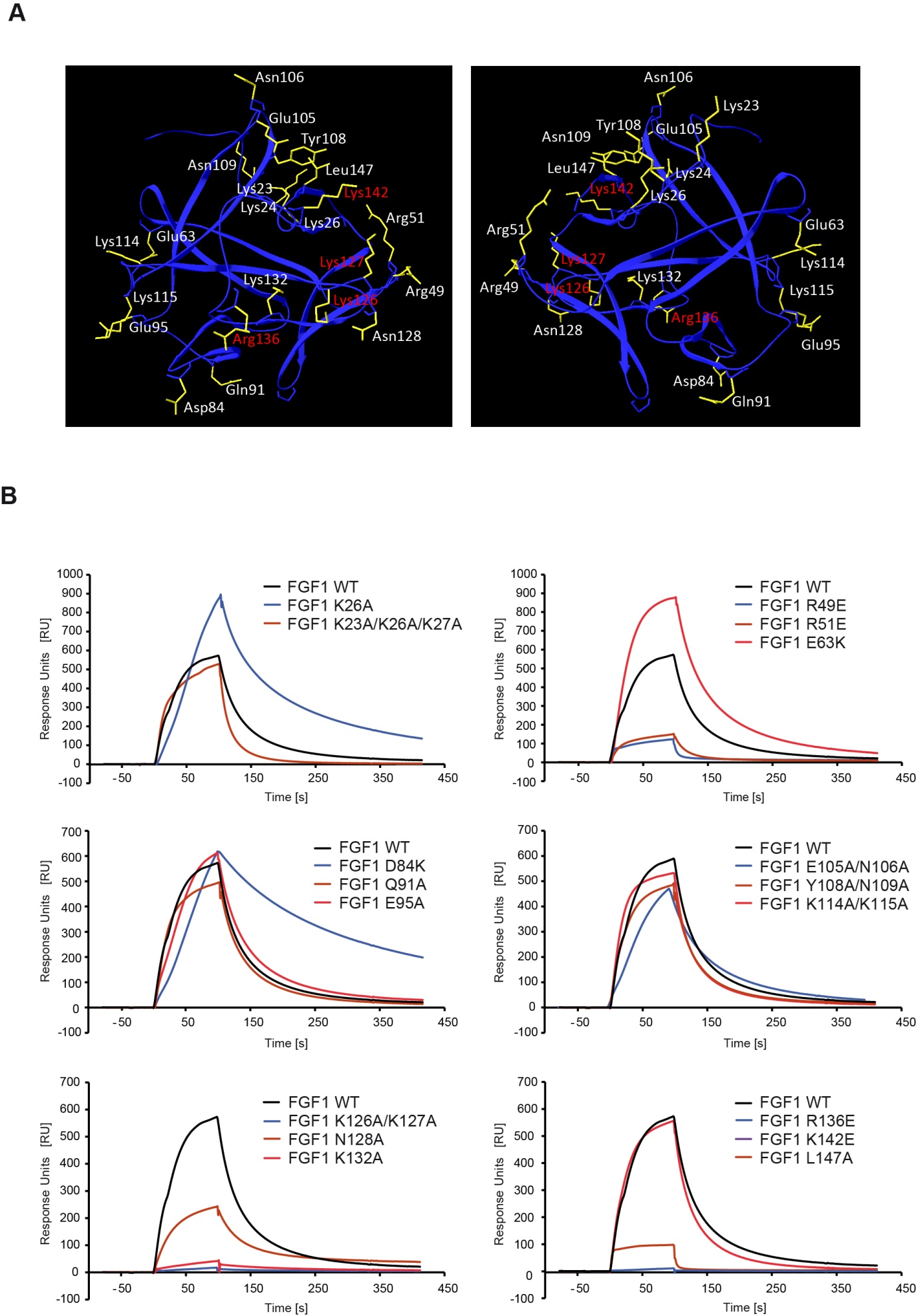
**

**Figure S4. Mutational analysis of FGF1 binding to nucleolin.** A) FGF1 residues potentially involved in binding with protein partners identified by bioinformatic analysis using the following web servers: meta-PPISP [36], ConSurf [37] and SWAKK [38]. Residues of FGF1 involved in nucleolin binding are marked with red. B) SPR measurements of interaction between 17 FGF1 mutants and the C-terminal fragment of nucleolin. Nucleolin-C was immobilized on a CM sensor chip at the level of ~5790 RU. Wild-type FGF1, as well as its mutational variants, were injected on the chip as analytes at concentrations of 654 nM.
