# Supplementary material for: Nucleolin Regulates Phosphorylation and Nuclear Export of Fibroblast Growth Factor 1 (FGF1)
Source: PLoS One. 2014 Mar 4;9(3):e90687. doi: 10.1371/journal.pone.0090687 (PMC3942467; doi:10.1371/journal.pone.0090687)
Supplement: Figure S5 — Elution profiles of FGF1. (DOCX) [file pone.0090687.s005.docx]

**Figure S5.**

**
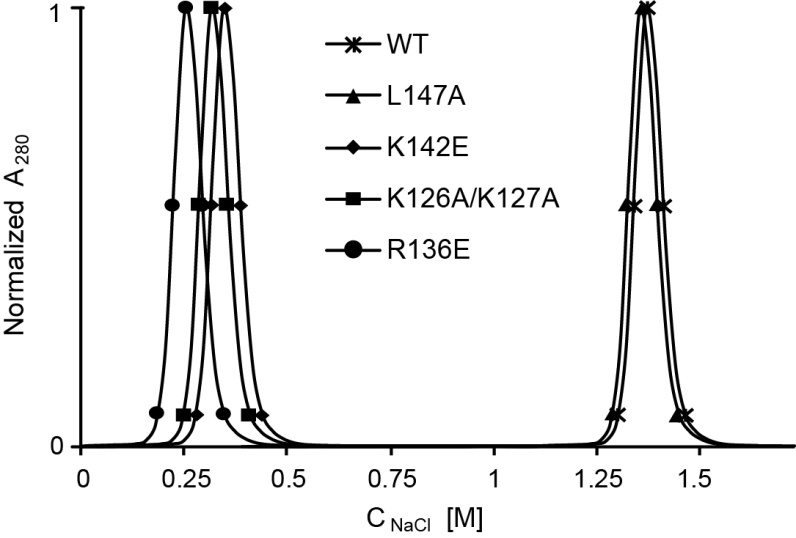
**

**Figure S5.** Elution profiles of FGF1 and its mutants from a Heparin-Sepharose column with a linear NaCl gradient.
