# Supplementary material for: Nucleolin Regulates Phosphorylation and Nuclear Export of Fibroblast Growth Factor 1 (FGF1)
Source: PLoS One. 2014 Mar 4;9(3):e90687. doi: 10.1371/journal.pone.0090687 (PMC3942467; doi:10.1371/journal.pone.0090687)
Supplement: Figure S6 — Non-overlapping siRNA sequences against nucleolin inhibits phosphorylation of FGF1 by PKCδ. (DOCX) [file pone.0090687.s006.docx]

**Figure S6.**

**
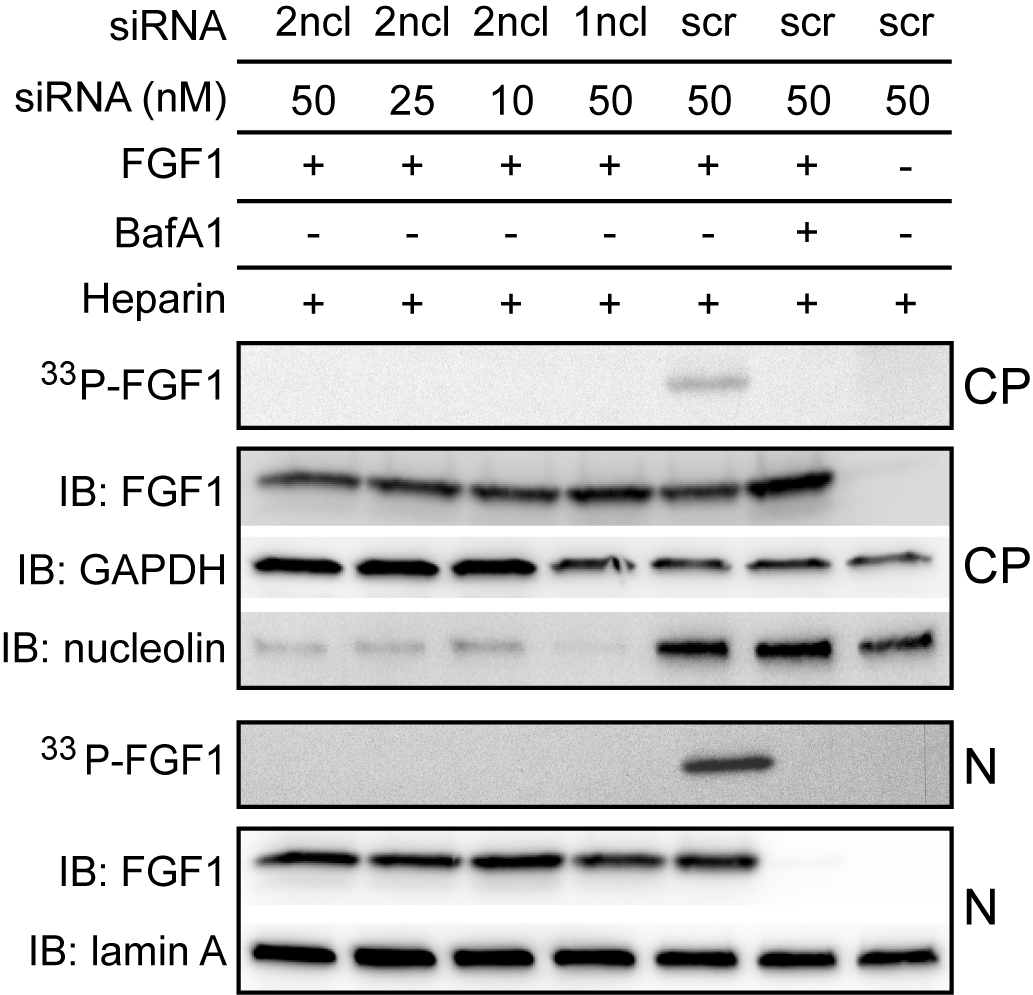
**

**Figure S6. Non-overlapping siRNA sequences against nucleolin inhibits phosphorylation of FGF1 by PKCδ.** U2OSR1 cells were transfected with siRNA against nucleolin obtained from Santa Cruz Biotechnology (sc-29230) (2ncl) or Qiagen (SI02654925) (1ncl), as indicated (non-overlapping sequences), serum starved for 24 h and labelled by [^33^P]phosphate, and thereafter stimulated with 100 ng/ml unlabelled, recombinant FGF1 in the presence of 10 U/ml heparin, and 10 nM BafA1 or 10 U/ml heparin alone, where indicated, for 6 h. The cells were fractionated into cytoplasmic (CP) and nuclear (N) fractions. FGF1 were extracted from the fractions by binding to Heparin-Sepharose and analyzed for phosphorylated FGF1 (^33^P-FGF1) by SDS-PAGE and fluorography, and immunoblotting (IB) to detect total FGF1. Fractions were also analyzed for marker proteins by IB as indicated.
