# Supplementary material for: Nucleolin Regulates Phosphorylation and Nuclear Export of Fibroblast Growth Factor 1 (FGF1)
Source: PLoS One. 2014 Mar 4;9(3):e90687. doi: 10.1371/journal.pone.0090687 (PMC3942467; doi:10.1371/journal.pone.0090687)
Supplement: Figure S7 — Nucleolin is required for intracellular phosphorylation of full lenght and truncated forms of FGF1. (DOCX) [file pone.0090687.s007.docx]

**Figure S7.**

**
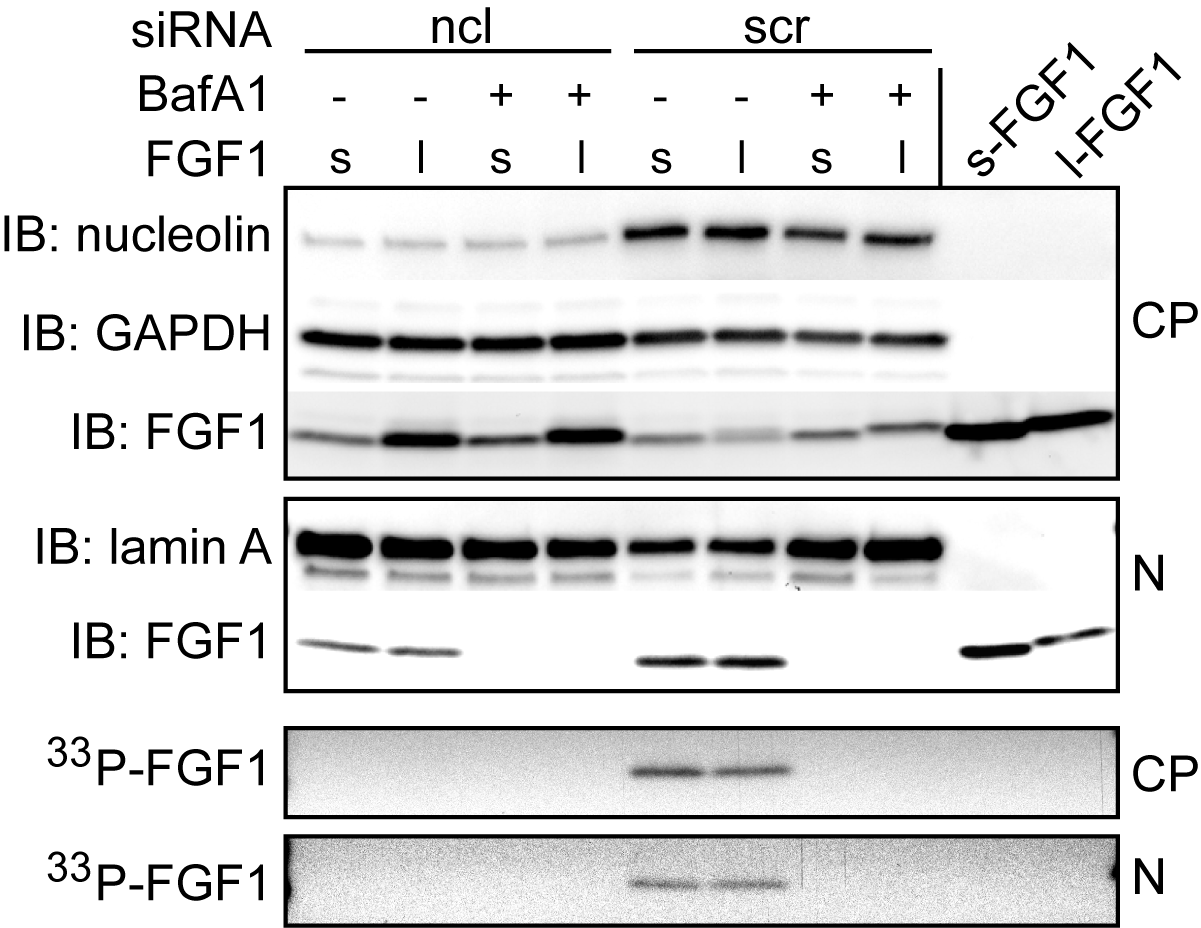
**

**Figure S7. Nucleolin is required for intracellular phosphorylation of full lenght and truncated forms of FGF1.** U2OSR1 cells were transfected with siRNA as indicated, serum starved for 24 h and labelled by [^33^P]phosphate, and thereafter stimulated with 100 ng/ml unlabelled, recombinant FGF1, either full-length (long, l-FGF1) or the truncated version (short, s-FGF1), in the presence of 10 U/ml heparin for 6 h. 10 nM BafA1 was added where indicated. The cells were lysed and fractionated into cytoplasmic (CP) and nuclear (N) fractions. Samples of the fractions were analysed by SDS-PAGE and immunoblotting (IB) as indicated. A sample of l-FGF1 and s-FGF1 was loaded directly on each gel (10 ng each on the CP gel and 5 ng each on the N gel, in lanes to the right) to indicate their difference in molecular weight/migration in SDS-PAGE. For the remainder of CP and N fractions, FGF1 was extracted by adsorption to Heparin-Sepharose and analyzed by SDS-PAGE and fluorography to detect the *in vivo* phosphorylation of FGF1 (^33^P-FGF1). As can be discerned by their mobility in SDS-PAGE, only the short form of FGF1 is detectible in the nucleus and as a phosphorylated protein, indicating that the full-length FGF1 was truncated (by a BafA1 sensitive process) before translocation into the nucleus.
