# Supplementary material for: Nucleolin Regulates Phosphorylation and Nuclear Export of Fibroblast Growth Factor 1 (FGF1)
Source: PLoS One. 2014 Mar 4;9(3):e90687. doi: 10.1371/journal.pone.0090687 (PMC3942467; doi:10.1371/journal.pone.0090687)
Supplement: Figure S8 — Nucleolin does not influence phosphorylation of FGF1 by PKCδ in vitro . (DOCX) [file pone.0090687.s008.docx]

**Figure S8.**

**
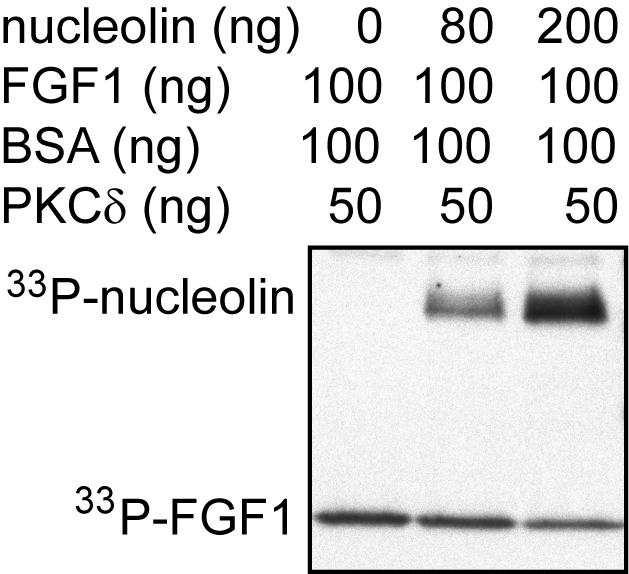
**

**Figure S8. Nucleolin does not influence phosphorylation of FGF1 by PKCδ *in vitro*.** Mixtures of varying amounts of recombinant nucleolin-C, FGF1, bovine serum albumin (BSA), and active PKCδ, as indicated, were incubated with [γ-^33^P]ATP for 30 min at 30ºC. The protein mixtures were then analyzed by SDS-PAGE and fluorography to detect phosphorylated proteins that were identified by their molecular weight.
