# Supplementary material for: Nucleolin Regulates Phosphorylation and Nuclear Export of Fibroblast Growth Factor 1 (FGF1)
Source: PLoS One. 2014 Mar 4;9(3):e90687. doi: 10.1371/journal.pone.0090687 (PMC3942467; doi:10.1371/journal.pone.0090687)
Supplement: Table S1 — Kinetic parameters of FGF1 and FGF2 binding to recombinant nucleolin-C. (DOCX) [file pone.0090687.s009.docx]

**Table S1. Kinetic parameters of FGF1 and FGF2 binding to recombinant nucleolin-C.**

| Protein | Component | Peak position k_a_ [M^-1^s^-1^] | Peak position k_d_ [s^-1^] | Peak position K_D_ [M] | Weight [%] |
| --- | --- | --- | --- | --- | --- |
| FGF1 | 1 | 1.7563×10^4^ | 7.0172×10^-4^ | 3.9955×10^-8^ | 20.4440 |
|  | 2 | 1.7418×10^6^ | 8.3130×10^-1^ | 4.7727×10^-7^ | 65.6451 |
| FGF2 | 1 | 1.7375×10^4^ | 3.8465×10^-4^ | 2.2138×10^-8^ | 43.4307 |
|  | 2 | 3.5404×10^6^ | 4.4315×10^-1^ | 1.2517×10^-7^ | 39.8266 |

FGF1 and FGF2 proteins were injected on a CM4 chip with immobilized nucleolin-C (immobilization level ~540 RU) at increasing concentrations (10-320 nM). The obtained sensorgrams were analyzed by Rigdeview Diagnostics AB (Uppsala, Sweden) and Interaction Maps were generated. Kinetic parameters and affinities were calculated on the basis of peak contribution of separate interaction components. Weight was calculated on the basis of single components contribution to the overall binding event.
